# Ignoring Imperfect Detection in Biological Surveys Is Dangerous: A Response to ‘Fitting and Interpreting Occupancy Models'

**DOI:** 10.1371/journal.pone.0099571

**Published:** 2014-07-30

**Authors:** Gurutzeta Guillera-Arroita, José J. Lahoz-Monfort, Darryl I. MacKenzie, Brendan A. Wintle, Michael A. McCarthy

**Affiliations:** 1 School of Botany, University of Melbourne, Parkville, Victoria, Australia; 2 Proteus Wildlife Research Consultants, Outram, New Zealand; Utah State University, United States of America

## Abstract

In a recent paper, Welsh, Lindenmayer and Donnelly (WLD) question the usefulness of models that estimate species occupancy while accounting for detectability. WLD claim that these models are difficult to fit and argue that disregarding detectability can be better than trying to adjust for it. We think that this conclusion and subsequent recommendations are not well founded and may negatively impact the quality of statistical inference in ecology and related management decisions. Here we respond to WLD's claims, evaluating in detail their arguments, using simulations and/or theory to support our points. In particular, WLD argue that both disregarding and accounting for imperfect detection lead to the same estimator performance regardless of sample size when detectability is a function of abundance. We show that this, the key result of their paper, only holds for cases of extreme heterogeneity like the single scenario they considered. Our results illustrate the dangers of disregarding imperfect detection. When ignored, occupancy and detection are confounded: the same naïve occupancy estimates can be obtained for very different true levels of occupancy so the size of the bias is unknowable. Hierarchical occupancy models separate occupancy and detection, and imprecise estimates simply indicate that more data are required for robust inference about the system in question. As for any statistical method, when underlying assumptions of simple hierarchical models are violated, their reliability is reduced. Resorting in those instances where hierarchical occupancy models do no perform well to the naïve occupancy estimator does not provide a satisfactory solution. The aim should instead be to achieve better estimation, by minimizing the effect of these issues during design, data collection and analysis, ensuring that the right amount of data is collected and model assumptions are met, considering model extensions where appropriate.

## Introduction

Species occupancy is a state variable widely used in ecology. It can be defined as the proportion of sites where the target species is present (or in terms of the underlying probability), and is relevant to monitoring programs and the study of species distributions. Models that allow its estimation while simultaneously accounting for imperfect detection are available and have become increasingly used over the past decade [1]–[4]. The key to these models is describing the data as the result of two linked processes: the state process (where the species occurs) and the detection process (how the species is detected at sites where present). Given this structure, models of this type are often referred to as ‘state-space models’ or ‘hierarchical models’ [5], a terminology that we adopt here. Imperfect detection is a widely recognized problem in ecological surveys [6], including those for sessile species [7], [8]. If not accounted for, imperfect detection can bias estimators of occupancy and habitat relationships [3], [9]–[11] and the underlying processes driving occupancy dynamics [1], [12], [13].

In a paper in this journal [14], Welsh, Lindenmayer and Donnelly (hereafter WLD) question the usefulness of hierarchical occupancy models after reporting results of simulations and theoretical calculations using the basic model in [2]. While assessing the performance of such models is important, we feel that WLD do not provide a representative assessment of the limitations and benefits of hierarchical occupancy models, and we note that some of their analyses appear to contain errors that have implications for some of their statements regarding estimator quality. In particular, we believe that WLD's key conclusion that ‘ignoring detection can actually be better than trying to adjust for it' is incorrect and may encourage poor practice in ecological data analysis. Here we present our view on the issues raised by WLD and re-examine their results.

WLD support their criticisms of hierarchical occupancy modelling by stating that these models lead to boundary estimates, “multiple solutions” and imprecise estimators of occupancy and detectability if the sample size is small. While we agree that estimator quality deteriorates with decreasing sample size, which is true for any type of statistical model, this does not justify general claims about lack of utility of hierarchical occupancy models. WLD select a few scenarios to justify their argument that disregarding detectability can be a better approach than explicitly modelling it. Using a more comprehensive analysis, including additional parameter values and methods of assessing the performance of the two approaches, we will demonstrate the true value of hierarchical occupancy models and how they outperform estimators that ignore detectability.

Despite suggestions by WLD to the contrary, the performance of single-species single-season occupancy models has been previously evaluated in the literature. For instance, in presenting the model, [2] assessed the models via simulations; [15]–[20] consider the precision of the estimators to address survey design, how to allocate survey effort optimally, and how to determine the sample size required to obtain meaningful results; [21] explores the problems of identifiability when detectability is heterogeneous; [22] considers the value of sampling with replacement in studies based on spatial replication within sites. Hierarchical occupancy models can lead to estimates at the boundary of the parameter space (occupancy estimates equal to one) when the sample size is small. WLD find these boundary estimates surprising, however such estimates were already mentioned in [2], while [17], who evaluate model performance under small sample size, derive the conditions fulfilled by data sets that lead to boundary estimates under the constant model. The references above demonstrate that there has been performance evaluation of these methods in the literature, although we acknowledge that further work in this area can be valuable.

Before moving to more specific comments in the next section, we clarify that, contrary to the assertion by WLD, [1] do not recommend studying changes in occupancy instead of abundance as a general rule. They note that occupancy and abundance are alternative state variables, the choice of which depends on ensuring that the results of monitoring are meaningful and hence on the objectives of the program (see also [6]). Occupancy is a reduced version of abundance (i.e. occupancy probability is the probability that abundance is greater than zero). While species occupancy might sometimes be sufficiently informative [23], we agree that this is not necessarily always the case. If one truly desires abundance estimates, then one should not use occupancy estimation methods. However, we note that issues of detectability and the requirement that suitable data are collected are just as relevant when estimating abundance (e.g., see [24]–[26]). Here we do not enter into the discussion of state variable choice any further, which, although interesting, is a different topic. Our focus in this paper is on addressing the criticisms regarding ‘fitting and interpreting occupancy models’ raised by WLD. Hence our premise is that species occupancy is the state variable of interest.

## Methods

We make five main points, which are supported by evidence from simulation results and mathematical derivations. Following WLD, we ran simulations for a scenario (hereafter Scenario A1) where occupancy was 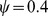 for all sites and detection probability was 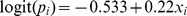, with covariate 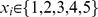, which corresponds to detection probabilities ranging 0.422–0.638 (in WLD the covariate represents years since plantation in surrounding habitat and the target species are woodland birds). We assumed the same number of sites for each value of the covariate 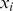. For comparison, we added a second scenario (hereafter Scenario A2) with higher occupancy and lower detectability: 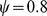 and 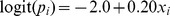 (i.e. 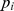 ranging 0.142–0.269). WLD also considered the case 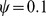 (with detection probability as for A1) but re-analysing that scenario does not change the main points we make subsequently, so we do not consider those analyses further; given sparse observations in this case, the models will perform poorly unless the sample size is relatively large [17].

We simulated 5000 data sets per scenario, and fitted hierarchical occupancy models with the package unmarked v0.9–9 [27] in R [28], which obtains maximum-likelihood estimates via numerical optimization. Unmarked differs from the R package VGAM used by WLD [29] in that it has been specifically developed for fitting hierarchical occupancy models (among others) rather than a more general family of models. Following WLD, we fitted the model with the covariate in both the occupancy and detection components, i.e. 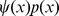, although more generally we suggest that one should consider some form of model selection technique to identify which covariates provide the best description of the available data. We also fitted standard logistic regression models (i.e. occupancy models assuming perfect detection, hereafter ‘naïve occupancy models’) using the R function *glm*. We again allowed occupancy to be a function of the covariate, i.e. 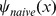. We fitted the naïve models to two sorts of data sets: first only considering a single replicate survey per site, then considering the same sampling effort as in the corresponding hierarchical model, but collapsing the data to a single detection/non-detection record per site (i.e. considering the species detected at a site if it was detected in any of the surveys). Regarding sample size, we evaluated all combinations of *S* = 55, 110 or 165 sites and *K* = 2, 3, 4 or 5 replicate surveys per site. Note that, despite mentioning four sample size cases, WLD only report results for the smallest sample size they assessed (i.e. *S* = 55 and *K* = 2). Following WLD, we treated fitted values greater than 0.9999 as one, and those smaller than 0.0001 as zero. We include an additional set of simulations that explores the entire parameter space, assuming constant occupancy and detectability (details in Appendix S2).

Following WLD, we ran a set of simulations in which detectability at each site within a covariate category was a random variable rather than constant (but the hierarchical model fitted still assumed that detectability was constant within each category, following a logistic regression as above, i.e. 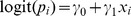). WLD present this as a scenario where detectability is a function of abundance but it can be interpreted more generally as any scenario where detectability is heterogeneous amongst sites. We ran this set of “abundance” simulations using both the same and different parameters as WLD (details are given in the relevant section below; we will refer to these simulations as Scenarios B1, B2 and B3). See Table 1 for a summary of all simulated scenarios. To corroborate our simulations, we further assessed these three scenarios by solving the expected estimating equations (as in WLD's ‘theoretical results’; details in Appendix S3). This method provides information about the asymptotic bias of the estimators, which we also explored in detail for a wide range of heterogeneity scenarios (from none to extreme), assuming a single covariate category for simplicity.

**Table 1:** Simulated scenarios (marked with asterisk * those also tested by WLD).

	Occupancy probability	Detection probability
Scenario A1*	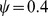	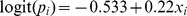 (i.e. 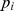 in 0.422–0.638)
Scenario A2	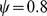	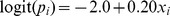 (i.e. 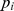 in 0.142–0.269)
Scenario B1*	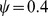	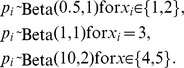
Scenario B2	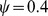	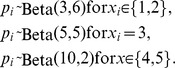
Scenario B3	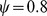	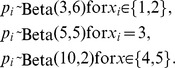

For all scenarios we tested all the combinations of the following sampling sizes: 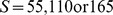 sites and 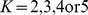 replicate surveys per site. The beta distributions below are plotted in Figure 4.

## Message 1: Boundary estimates and multiple solutions are not as great a problem as implied by WLD

WLD state that hierarchical occupancy models often lead to boundary estimates (i.e. estimates that take value 0 or 1) and suffer from multiple solutions. Boundary estimates are only a problem when the sample size is small (relative to the sparseness of the data) and occupancy estimates of 1 can be obtained even if the true underlying occupancy is low [17]. When the sample size is large, occupancy estimates on or close to 1 can still be obtained, but these are likely to faithfully correspond to a true high level in species occupancy. Although there is no doubt that the performance of the estimators worsens as the sample size decreases, we believe that the claims made by WLD regarding boundary estimates and multiple solutions are overstated for the following reasons:

### 1-1) ‘All one’ occupancy estimates are not a general problem

WLD present the system of equations that the maximum-likelihood estimates (MLEs) satisfy and support their claim that boundary estimates are often a problem in hierarchical occupancy models by observing that all sites being occupied (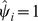 for all sites 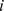) is always a solution for *one* of the equations in the system (eq (4) in [14]). It is, however, important to note that such a solution by itself does not represent a stationary point in the likelihood function (i.e., a point where all partial derivatives are zero, that is, where the surface is locally flat). For the point to be stationary, all the equations in the system need to be satisfied. Even then, the solution does not necessarily correspond to the maximum-likelihood estimate (and may in fact be a much less likely solution).

**Figure 1:**
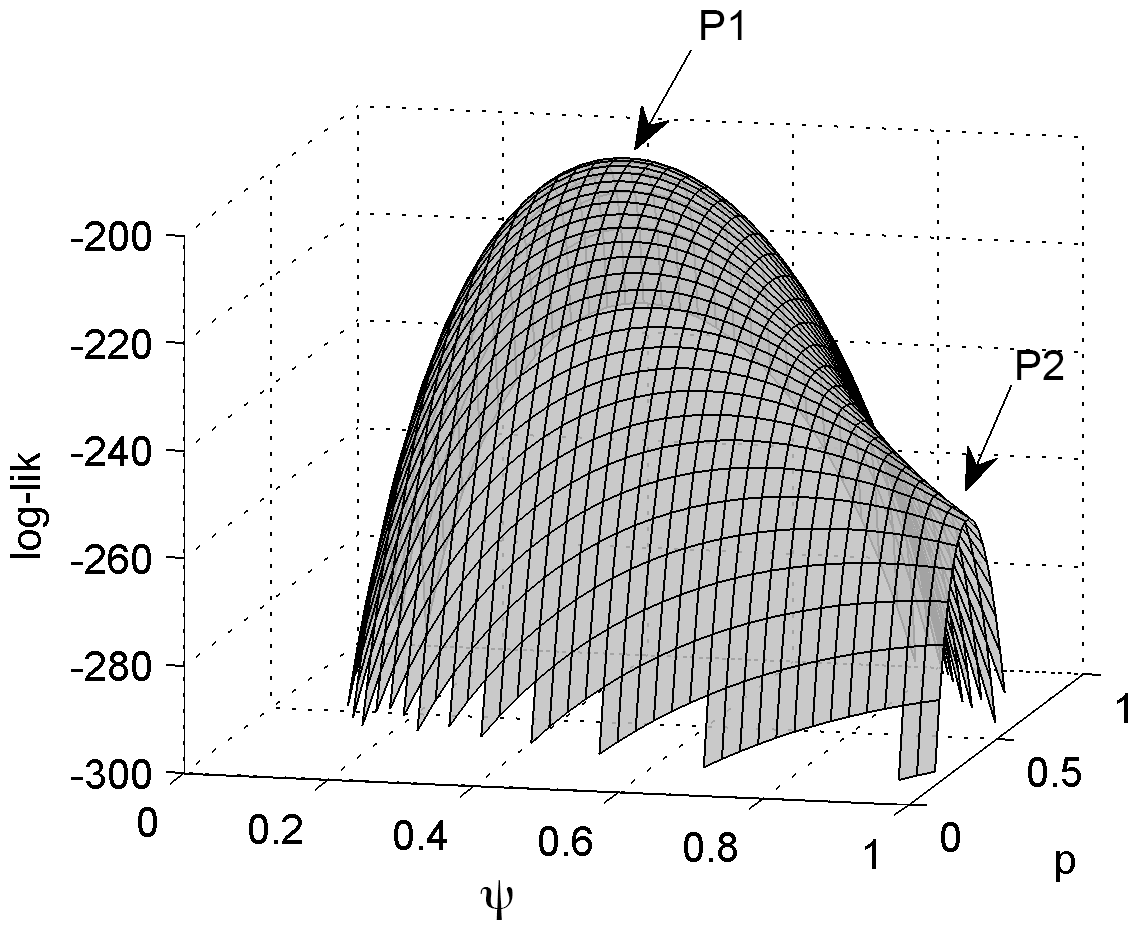
Log-likelihood surface displaying the maximum-likelihood estimate (P1) and a saddle point at the boundary 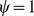 (P2). This example corresponds to a constant hierarchical occupancy model and a data set where *S* = 200 sites, *K* = 2 replicate visits, *S_d_* = 80 sites with detection and *d_T_* = 134 detections. P1 is located at {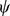 = 0.416, 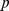 = 0.806} and P2 at {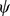 = 1, 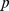 = 0.335}.

Consider for simplicity the model without covariates. Let 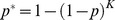 be the probability of detecting the species at least once at a site that is occupied, 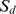 the total number of sites where the species is detected and 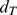 the total number of detections across all sites. The system of equations, which is obtained by differentiating the log-likelihood with respect to the parameters and equating to zero, indeed has a solution at 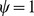 and 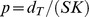. However, it can be shown that this solution corresponds to the maximum in the likelihood (and hence is the MLE) *only* when the following condition holds [17]: 
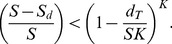
(1)When (1) does not hold, the solution above is a saddle point in the likelihood function, and not a maximum (Figure 1). The optimization algorithm used to obtain the estimates should not have particular problems locating the true maximum of the function, which is at 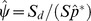 and 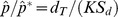, even if there is another stationary point (see more about dealing with multiple solutions in point 1-3 below). In our analysis, none of the 5000 simulations led to ‘all-one’ occupancy estimates in Scenario A1 when *S* = 55 and *K* = 2, or for larger sample sizes (Table 2b), while WLD only encountered 12 cases for the same scenario (Table 2a).

**Table 2:** Counts of different estimation results obtained when fitting hierarchical occupancy models to simulated data from Scenario A1.

(a) Results from WLD								
		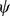						
		all 0	some 0	some 0&1	some 1	all 1	interior	total
*p*	all 0	0	0	0	0	0	0	0
	some 0	0	0	0	0	0	0	0
	some 0&1	0	0	9	0	0	0	9
	some 1	48	1	11	0	0	0	60
	all 1	62	0	0	0	0	0	62
	interior	10	0	21	57	12	4769	4869
	Total	120	1	41	57	12	4769	5000

In (a) results obtained by [14], in (b) results obtained in this study. In both cases estimates were categorized as 0 or 1 based on thresholds 0.0001 and 0.9999 respectively. Sample size *S* = 55 sites and *K* = 2 replicate surveys. Model fitted 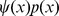.

### 1-2) ‘All zero’ occupancy estimates are not possible unless there are no detections

The simulation results presented by WLD for Scenario A1 show occupancy estimates 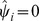 (for all sites *i*) in data sets *with* detections (120 out of 5000 simulations; Table 2a). WLD state that this ‘*seems strange*’. Indeed, such results cannot be maximum-likelihood estimates, and must be errors. This can be immediately seen by looking at the first of the estimating equations (eq (4) in [14]), which is only equal to zero when 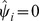 (for all sites *i*) if there are no detections at any site (i.e. 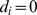 for all sites *i*). As expected, we did not obtain such estimates (Table 2b). In all of our simulations, the species was detected at ≥4 sites, and at least one of the occupancy estimates was greater than 0.16; that is, there was always at least one estimate that was well above zero. We verified that very similar results are obtained with program PRESENCE [30]. The results presented by WLD, which contradict theory, point to problems either with their data simulation or with the R package they used for model fitting (VGAM). We believe it was most likely the latter given the difficulties to achieve convergence that we experienced when testing the VGAM package in a subset of our simulations with *K* = 2.

### 1-3) Difficulties of dealing with multiple solutions are overstated

WLD claim that obtaining multiple solutions to the system of likelihood equations is a problem in hierarchical occupancy models. To evaluate the extent to which multiple solutions are indeed a problem for model fitting, we reran our Scenario 1 simulations for the smaller sample size (*S* = 55 and *K* = 2), fitting each data set multiple times (20 each) with different randomly chosen starting values and examined the estimates obtained with each of them.

In 98.5% of the simulations, unmarked found the maximum-likelihood estimates at the first attempt using its default values. Note that WLD also acknowledge that their fitting procedure usually returned the MLE with its default settings. This confirms that the difficulties of dealing with multiple solutions are not as great as conveyed by some of the statements made by WLD.

The optimization algorithm used by unmarked tended to consistently find the MLE (98.7% of the attempts) as long as the initial values given for the regression coefficients were kept within reasonable values (e.g. choosing values in [−0.5,0.5]). The optimization algorithm was more prone to stop at an estimate that was at the boundary (i.e. with at least one value 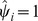) and that was not the MLE (i.e. had smaller likelihood) only when we allowed for large initial values for the regression coefficients (e.g. choosing values in [−3,3]). Hence, to reduce the chances of ending at a point different from the MLE, one should avoid using extreme starting values, which may make the optimization algorithm start (and get stuck) at the boundary. In any case, fitting the model with multiple starting values is always recommended to ensure that the MLE has been located (and not a local maximum). Using multiple starting values is good practice that is not unique to hierarchical occupancy models, but rather should be routinely considered whenever estimating parameters by numerical maximum-likelihood techniques.

## Message 2: By ignoring imperfect detection, a different metric is estimated. This metric can be derived from the hierarchical model

**Figure 2:**
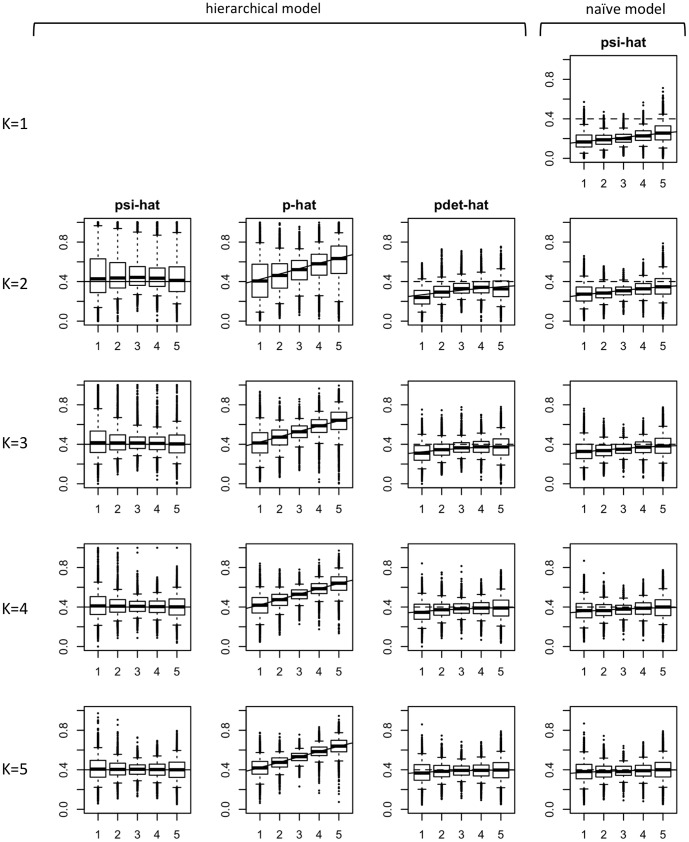
Simulation results of fitting hierarchical and naïve occupancy models to 5000 data sets from Scenario A1 with 55 sites. The first three columns correspond to the hierarchical model: in column 1 estimates of occupancy probability 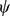 (‘psi-hat’), in column 2 estimates of the conditional single-survey detection probability 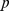 (‘p-hat’) and in column 3 estimates of the unconditional detection probability after *K* surveys 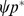 (‘pdet-hat’). Column 4 presents the estimates for the naïve model that assumes perfect detection. Rows represent increasing number of replicate surveys per site, from *K* = 1 to *K* = 5. Where 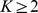 the naïve model was fitted to data collapsed to a single record per site (1 if species detected at least once, 0 otherwise). In this particular scenario (also presented by [14]) the imprecision in the hierarchical model is large compared to the bias in the naïve model. The true occupancy was 0.4, and the true detection probability increased with the value of the x-variable. In each figure a solid line represents true values. For reference, in columns 3 and 4 a dashed line represents the true occupancy probability.

**Figure 3:**
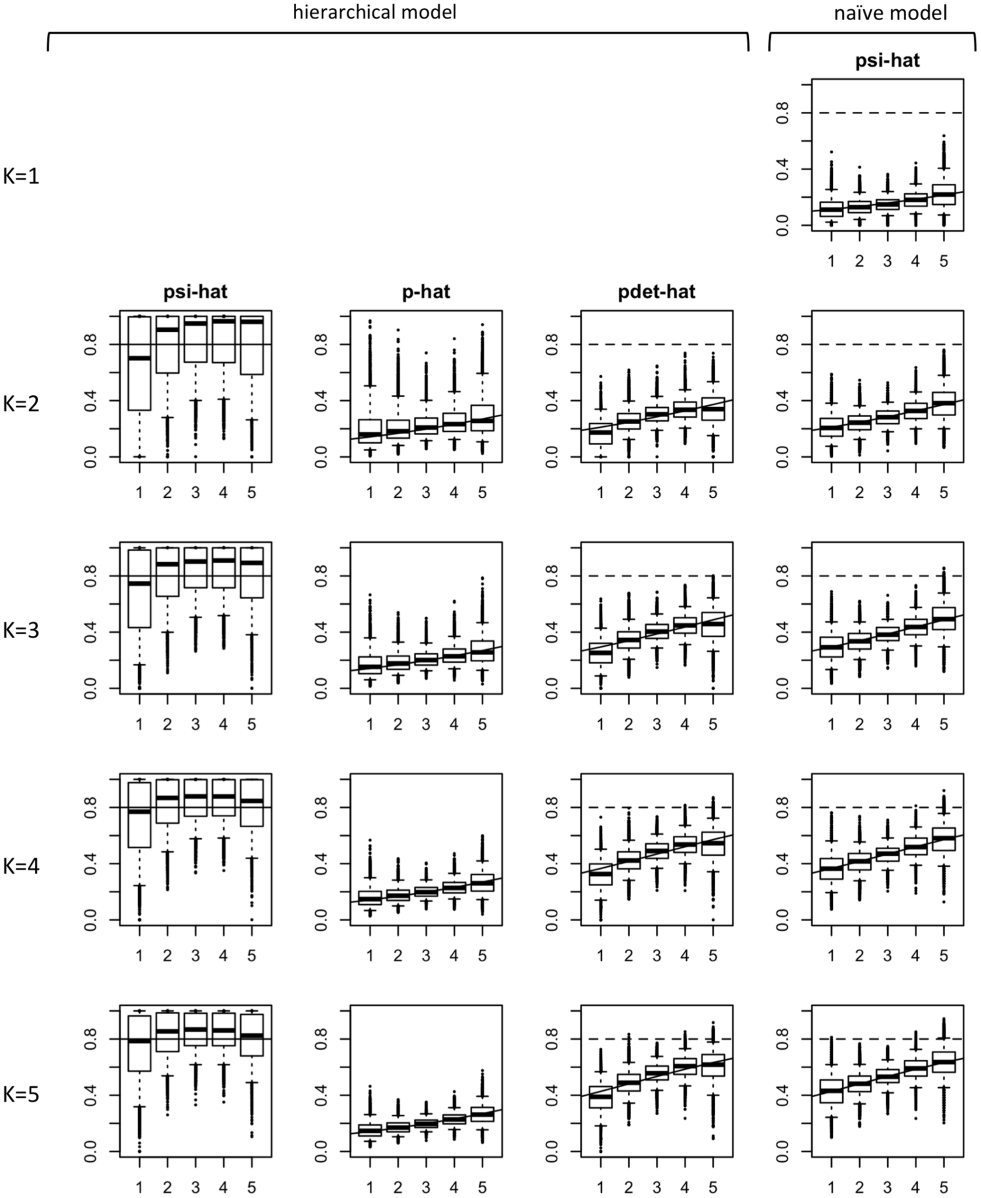
Simulation results of fitting hierarchical and naïve occupancy models to 5000 data sets from Scenario A2 with 55 sites. For details in figure arrangement see Figure 2; here the true occupancy was 0.8. In this example the hierarchical model clearly outperforms the naïve model, which is greatly biased. A comparison of the estimates for *K* = 2 illustrates with those in Figure 2 illustrates how the naïve model can produce the same estimates for very different scenarios.

When imperfect detection is disregarded, the metric being estimated is no longer species occupancy (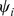). Occupancy and detection are confounded so the model estimates instead the *product* 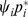, where 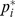
is the probability of detecting the species at a site where present given the total survey effort. Rather than estimating where the species is more or less likely to occur, the model estimates where the species is more or less likely to be detected (with the methods and effort employed). This is a problem of parameter identifiability, where the model cannot tease apart the state process and the observation process, and can also be interpreted as a situation where the estimation of species occupancy is biased by an unknown amount. It is worth noting that the hierarchical occupancy model can also estimate this metric (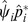) based on its estimates of occupancy 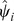 and detection 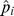, with 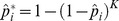. Figures 2 and 3 show how the estimates derived for this quantity with the hierarchical occupancy model (third column) are essentially the same as the estimates obtained when fitting the naïve model (fourth column).

## Message 3: Accounting for imperfect detection provides a more reliable estimator of occupancy, which honestly captures the uncertainty

When interpreted as an estimator of species occupancy, the naïve model is biased whenever overall detection is imperfect (i.e. 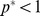; see column 4 in Figures 2 and 3; also Figures S1.1–S1.6 in Appendix S1) [2], [3], [9], [10]. The hierarchical occupancy model provides instead an asymptotically unbiased occupancy estimator when model assumptions are met (column 1 in the same figures). However, its estimator is less precise. This is expected given that there is an additional source of uncertainty once imperfect detection is admitted [1], [15]. Hence, for small sample sizes and depending on the scenario, the hierarchical occupancy model may lead to an estimator with a mean square error (MSE) that is larger than that in the naïve estimator (see Table S2.1 in Appendix S2). However, as we show below, one cannot tell from the data whether one is in such a scenario, or in one where ignoring detectability implies a large bias (and hence MSE).

As we increase the number of survey sites *S*, the performance of the hierarchical model improves because its estimator becomes more precise. The naïve model also becomes more precise but, since it remains biased, its performance does not appreciably improve unless the bias is small. The naïve model provides an estimator that is not consistent, i.e., its MSE does not tend to zero as sample size *S* increases. The superiority of the hierarchical model in terms of MSE is thus more apparent as *S* increases (see Table S2.1 and MSE ratios in Table 3). In the naïve model, increasing the number of surveyed sites can in fact be detrimental: the confidence interval narrows around an incorrect estimate (since the estimator is biased). Apart from small MSE, a desirable property of estimators is to provide confidence intervals that have good coverage, that is, confidence intervals that tend to contain the true parameter value (e.g. 95% of the time when working with 95% confidence intervals). Due to the fact that the naïve estimator is biased, its coverage can be very poor (see Table S2.2 in Appendix S2). Hence the naïve model can provide misleadingly precise estimates that are far from the true occupancy value.

**Table 3:** Mean square error (MSE) for the occupancy estimator in the hierarchical/naïve models, and their ratio, obtained from simulations of (a) Scenario A1 and (b) Scenario A2 (see Table 1 for details).

(a) Scenario A1			
	*S* = 55	*S* = 110	*S* = 165
*K* = 1	NA/0.042	NA/0.039	NA/0.038
*K* = 2	0.047/0.017 = 2.82	0.019/0.013 = 1.44	0.011/0.011 = 0.94
*K* = 3	0.017/0.011 = 1.62	0.007/0.007 = 1.08	0.004/0.005 = 0.84
*K* = 4	0.011/0.009 = 1.22	0.005/0.005 = 1.04	0.003/0.004 = 0.97
*K* = 5	0.010/0.009 = 1.10	0.005/0.005 = 1.04	0.003/0.003 = 1.02

(b) Scenario A2			
	*S* = 55	*S* = 110	*S* = 165
*K* = 1	NA/0.413	NA/0.411	NA/0.411
*K* = 2	0.075/0.271 = 0.28	0.052/0.267 = 0.19	0.043/0.268 = 0.16
*K* = 3	0.052/0.182 = 0.28	0.034/0.177 = 0.19	0.027/0.177 = 0.16
*K* = 4	0.038/0.124 = 0.31	0.025/0.119 = 0.21	0.019/0.118 = 0.16
*K* = 5	0.030/0.085 = 0.36	0.019/0.081 = 0.24	0.014/0.080 = 0.18

Simulations were run for a range of sample sizes, with *S* sites and *K* replicate surveys per site (5000 simulations per case). When 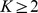, the naïve model was fitted to the data resulting from collapsing the detection/non-detection history into a single record per site (1 if species detected at least once, 0 otherwise). In the majority of these cases the performance of the hierarchical model was either comparable or considerably superior to that of the naïve model. A ratio <1 indicates that the MSE of the hierarchical model is smaller than in the naïve model.

WLD present Scenario A1 (*S* = 55 and *K* = 2) in making the case that modelling detectability is unnecessary because the bias induced by assuming perfect detection is relatively small compared to the reduced precision in the hierarchical model. Indeed, this particular scenario is one where the MSE for the estimator of the naïve model is smaller (Table 3a). However, it is crucial to remember that the naïve model could provide identical estimates to those arising from Scenario A1 for other occupancy scenarios that imply a much greater bias, where the hierarchical model clearly outperforms the naïve model. For instance, the estimates obtained when fitting the naïve model to Scenario A1 are essentially the same as those obtained in Scenario A2 when *K* = 2 (compare the corresponding plots in Figures 2 and 3; also Figures S1.2–S1.3 with Figures S1.5–1.6 in the Appendix S1 for larger sample size), but in the latter the true occupancy probability is 0.8 and hence the naïve estimator is substantially biased and has a much greater MSE (Table 3b). By looking at the naïve occupancy estimates alone we cannot tell how good or bad these estimates are, as the occupancy and detection processes are confounded (e.g., if the naïve occupancy is 0.24, is that because 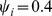 and 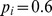 or 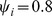 and 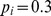
?). It is only when we partition these processes that we can understand whether imperfect detection is or is not an issue, and we can be confident that we are estimating species occupancy reliably. The naïve model estimator has poor coverage when detection is imperfect, that is, confidence intervals around estimates are unlikely to include the true occupancy value. We argue that it is better to be openly uncertain, than to be report a result that may be precise but wrong. Hence we believe that the hierarchical model provides an estimator more suitable to rely on.

## Message 4: Accounting for imperfect detection does not imply a need for increased sampling effort. Imperfect detection does

Accounting for imperfect detection does not necessarily require increased survey effort. However, it is necessary to collect survey data in such a way that the detection process can be modelled [10]. This can be done for instance by recording the observations gathered in multiple visits (as assumed here), the detections of multiple independent observers [1] or the detection times within a single visit [7], [31]. Another matter is that, for a given level of sampling effort per site, more sampling sites are needed to obtain good occupancy estimates when detectability is low. Simply put: poor detectability makes disentangling occupancy and detection processes harder. On the other hand, increasing the amount of survey effort per site (e.g. the number of replicate visits per site) reduces the problem of imperfect detection as the chances of missing the species at occupied sites decrease. For a given survey budget, there is a trade-off between visiting more sites and spending more survey effort per site [15]–[20], with the optimal effort allocation corresponding to relatively high levels of overall detection 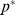 (around 0.85-0.95).

WLD present as a difficulty the need for “extra data collection […] to adjust for non-detection”. However, inconsistently, when fitting the naïve logistic model in their simulations, WLD use the data corresponding to the full sampling effort (collapsing the replicate records as we do). If WLD choose to associate the additional replicate surveys as a complication introduced by modelling detectability, then a fair comparison would have fitted the naïve model to the data from a single replicate survey per site. We include these results (denoted “*K* = 1”) as the first row in Table 3 and in our simulation results (Figures 2 and 3, and figures in Appendix S1) to illustrate the increased gap in performance between the naïve and hierarchical models when this approach is taken to fitting the naïve model. If the models are instead compared to data derived from the same sampling effort (as done by WLD), then the amount of sampling effort should not be used as an argument against the use of hierarchical models.

## Message 5: Hierarchical models are less biased than naïve models even if detection is a function of abundance

WLD's stated key result is that “when the detection process depends on abundance, the bias in the fitted probabilities can be of similar magnitude to the bias when the detection process is ignored, and this is very difficult to overcome”. They also point out that increasing the sample size (*S* or *K*) does not resolve the issue but instead makes estimates worse. The evidence for their conclusion comes from considering a single scenario using simulation and theoretical results. However, we have seen above that the hierarchical occupancy model behaves better than the naïve model in scenarios where detectability varies, and that could likewise be interpreted as cases in which detectability depends on abundance. So, what is the cause of this incongruence? And how can we explain the counterintuitive result that increasing sample size makes things worse?

**Figure 4:**
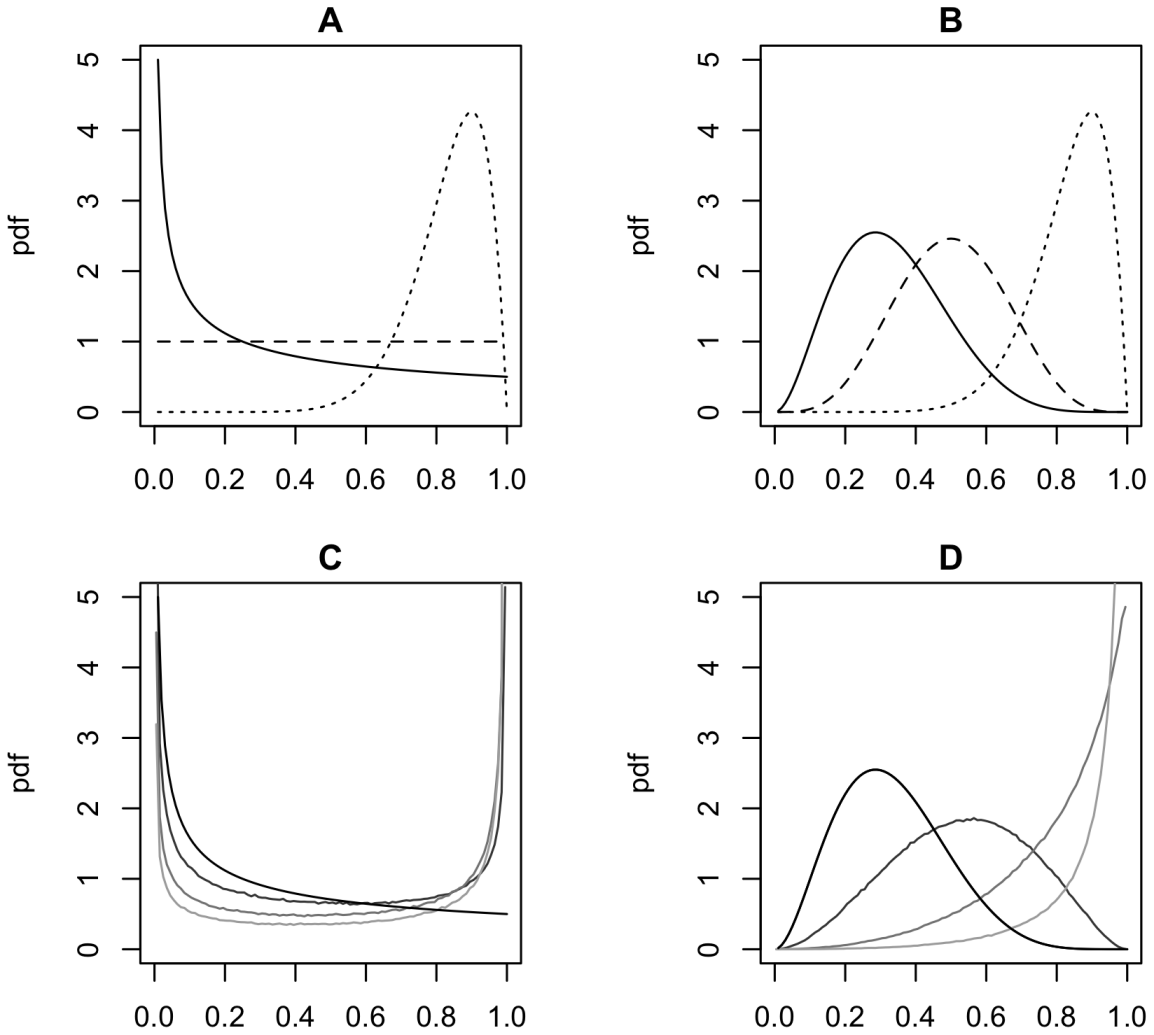
Beta distributions used to generate detectability in the “abundance” scenarios for the different covariate categories. Lines correspond to 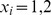 (solid), 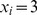 (dashed) and 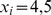 (dotted). Panel (a) displays the probability density functions (pdf) for the distributions used by WLD (Scenario B1) and panel (b) for the distributions used in our Scenarios B2 and B3. The distribution that WLD used for 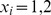 has considerable mass for detectability very close to zero: 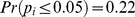. Panels (c-d) display the pdf of the probability of detecting the species in at least one of *K* surveys (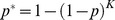) at sites 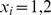 (from darker to lighter, lines correspond to 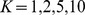).

A close inspection of the scenario simulated by WLD (hereafter Scenario B1) reveals the root of this contradiction. As in the previous example, occupancy was set constant for all sites (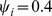 for all site *i*) and detectability varied for each covariate category 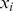, but here detectability values were generated as random variables from different distributions for each covariate category, hence introducing variation in the detection probability among sites (making the 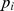
's vary). For their example, WLD chose the following distributions 
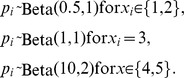
These distributions (Figure 4 a,b) represent a case in which detectability is relatively high for the two last covariate categories (90% of the mass in [0.636–0.967]; mean 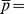
0.833 for 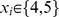) and can take any value for the other three category covariates, although it is more likely to be lower for 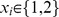 ([0.002–0.902]; mean 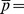
0.333) than for 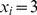 ([0.050–0.950]; mean 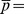
0.5). Importantly, for 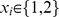 much of the probability mass is very close to zero (>22% of the “occupied” sites having detection probability <0.05: Figure 4a), while there is also a considerable chance that detection probability is high (i.e., approximately 22% of the distribution is >0.61). The distribution for the probability of at least one detection from the two surveys (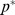) has a ‘bathtub’ shape with peaks near both 0 and 1 (Figure 4c). This rather extreme distribution implies that, at some occupied sites, the species is practically invisible to the survey methods, while at others its detection is almost guaranteed. When applying a model that does not allow for such heterogeneity, occupied sites with low detection probabilities are more likely to be regarded as unoccupied if the species goes undetected, particularly with only two surveys and if other sites have very high detection probabilities. This causes the negative bias observed by WLD in the estimation of occupancy for those two covariate categories, and hence the positive bias in the estimation of the slope in the occupancy regression. One cannot expect a model that assumes no heterogeneity to provide reliable inference about a species in the face of such extreme heterogeneity, even if the sample size is large. This is related to why WLD wrongly conclude that increasing the sample size (*S* or *K*) does not improve the performance of the hierarchical model. WLD observe that for this particular example the slope estimates “get slightly worse” as *K* increases, and “much worse” as *S* increases, by relating the quality of the estimator to the proportion of simulations that lead to a non-positive estimated slope. Indeed, since in these simulations the performance is dominated by the bias caused by having extremely low detectability at some sites in 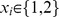, the improvement in precision due to an increase in the amount of data can lead to a greater proportion of simulations having positive slopes. However, WLD overlook that the bias itself also decreases (albeit slowly), and that therefore the quality of the estimator (measured in terms of MSE) indeed improves (Table 4). Estimator quality improves with *K*, which is intuitive as the model is ultimately unbiased when *K* is large enough so that the species is detected at all sites where present (unless *p* is exactly 0). Estimator quality also improves with *S* although some bias remains even for large *S* given that we are fitting a different detection structure than that which is used to generate the data. Our results also show that, even in this scenario, the hierarchical model outperforms the naïve model in terms of MSE (Table 4). The improvement is modest because the scenario is dominated by the fact that the species is virtually undetectable in a large fraction of the lower covariate category sites. As we show below, it is not surprising that WLD found that the biases were very similar in both the hierarchical and the naive model given the extreme fluctuations in detectability in the scenario evaluated, which we believe represents an unusual case.

**Table 4:** Mean square error (MSE) for the occupancy estimator in the hierarchical/naïve models, and their ratio, obtained from simulations of three “abundance” scenarios: (a) Scenario B1 from [14], (b) Scenario B2 and (c) Scenario B3.

(a) Scenario B1			
	*S* = 55	*S* = 110	*S* = 165
*K* = 1	NA/0.046	NA/0.040	NA/0.038
*K* = 2	0.028/0.032 = 0.87	0.018/0.025 = 0.72	0.017/0.023 = 0.73
*K* = 3	0.021/0.026 = 0.82	0.016/0.020 = 0.82	0.015/0.017 = 0.84
*K* = 4	0.019/0.023 = 0.86	0.015/0.017 = 0.88	0.013/0.014 = 0.90
*K* = 5	0.018/0.020 = 0.89	0.013/0.015 = 0.92	0.012/0.013 = 0.93

(b) Scenario B2			
	*S* = 55	*S* = 110	*S* = 165
*K* = 1	NA/0.041	NA/0.041	NA/0.041
*K* = 2	0.022/0.022 = 0.97	0.013/0.021 = 0.60	0.009/0.020 = 0.44
*K* = 3	0.014/0.016 = 0.93	0.008/0.013 = 0.63	0.006/0.012 = 0.51
*K* = 4	0.012/0.012 = 0.96	0.007/0.009 = 0.71	0.005/0.008 = 0.61
*K* = 5	0.011/0.011 = 0.98	0.006/0.007 = 0.80	0.004/0.006 = 0.70

(c) Scenario B3			
	*S* = 55	*S* = 110	*S* = 165
*K* = 1	NA/0.148	NA/0.156	NA/0.158
*K* = 2	0.029/0.068 = 0.42	0.024/0.072 = 0.34	0.020/0.071 = 0.28
*K* = 3	0.019/0.038 = 0.49	0.016/0.040 = 0.39	0.013/0.038 = 0.34
*K* = 4	0.014/0.025 = 0.58	0.011/0.024 = 0.46	0.009/0.023 = 0.40
*K* = 5	0.011/0.017 = 0.66	0.009/0.016 = 0.54	0.007/0.015 = 0.46

Simulations were run for a range of sample sizes, with *S* sites and *K* replicate surveys per site (5000 simulations per case). When 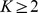, the naïve model was fitted to the data resulting from collapsing the detection/non-detection history into a single record per site (1 if species detected at least once, 0 otherwise). The hierarchical model outperforms the naïve model, being clearly superior in the third example.

**Figure 5:**
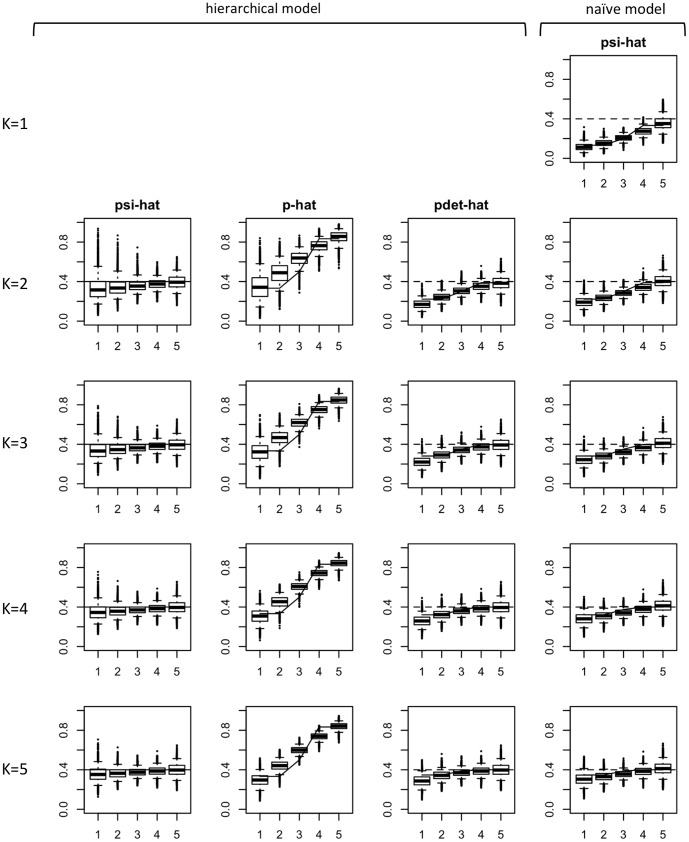
Simulation results of fitting hierarchical and naïve occupancy models to 5000 data sets from Scenario B2 with 165 sites. For details in figure arrangement see Figure 2. This example shows that, even if detectability is heterogeneous, the hierarchical model has smaller bias and that this bias is reduced with the sample size.

So, what would happen if we consider a different and plausible scenario? Let us consider an example where
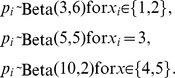
These distributions have the same mean for each covariate category as in the previous scenario (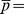
0.333, 0.5 and 0.833, respectively) and, although substantial, have less extreme levels of heterogeneity (Figure 4 b,d). Simulations with these detectability distributions and occupancy probability 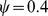 (Scenario B2) or 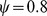 (Scenario B3) show that the hierarchical model clearly outperforms the naïve model in these examples (Table 4), even given that the linear model fitted to the detection component is misspecified relative to what was actually used for data generation. Although there is some residual bias in the hierarchical model (induced by small sample size and the fact that the model is asymptotically biased due to the misspecification of the detection component), the bias is noticeably reduced (Figure 5; see other figures in Appendix S1). The naïve model continues to be substantially biased due to its ignorance of imperfect detection. Our simulation results are corroborated by our theoretical evaluation (Appendix S3). WLD restricted their theoretical results to a single scenario (Scenario B1), but our extended evaluation shows that the asymptotic bias can indeed be substantially larger in the naïve model than in the hierarchical model even when detectability is heterogeneous (Table S3.1 and Figure S3.1).

**Figure 6:**
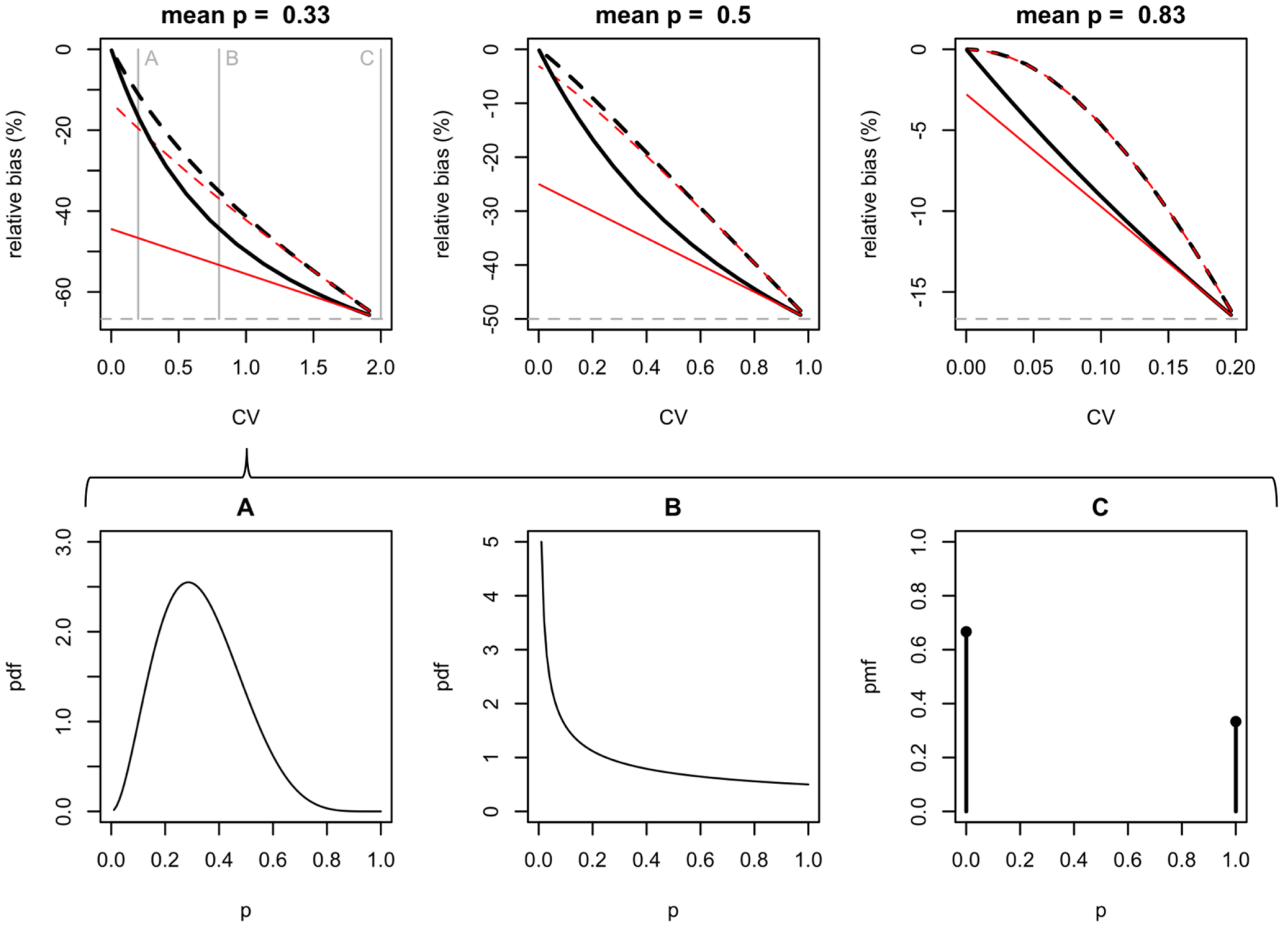
Asymptotic bias of the naïve and hierarchical occupancy estimators as a function of heterogeneity in detectability. In the data-generating model, occupancy is constant and detectability at each site is drawn from a single distribution 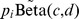. In the fitted model both occupancy and detectability are assumed constant across sites (i.e. heterogeneity is not modelled). Heterogeneity is expressed in the x-axis as the coefficient of variation of the distribution (CV). Black thick lines represent the hierarchical model and red thin lines the naïve model (solid lines for *K* = 2 and dashed lines for *K* = 5; horizontal grey lines correspond to a naïve model where *K* = 1). In extreme heterogeneity conditions (high CV such that detectability switches between 0 and 1) both models lead to the same bias. For more realistic scenarios, where heterogeneity is still substantial, the hierarchical model has lower asymptotic bias. The hierarchical model is asymptotically unbiased in the absence of heterogeneity (i.e. CV = 0). Plots in the lower row (A–C) illustrate the heterogeneity in detectability represented by three different CVs when mean detectability is 0.33. Note that the relative asymptotic bias is independent of occupancy probability.

The same conclusions can be drawn from our detailed exploration of a wide range of heterogeneity scenarios (from none to extreme), assuming a single covariate category (Figure 6). We derived the corresponding analytical expressions for the relative asymptotic bias of the occupancy estimator in the naïve and hierarchical models, which are
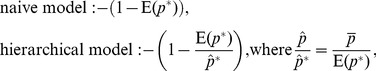
(2)with hats (∧) indicating estimates. We observe that, under extreme heterogeneity (i.e. detectability switching only between values 0 or 1), the asymptotic bias for both models is 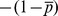. As heterogeneity decreases, the bias of the naïve model reduces but remains at 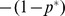 in the absence of heterogeneity. The bias of the hierarchical model decreases faster, reaching zero for the case without heterogeneity, and can be substantially lower that the bias in the naïve model for realistic heterogeneity scenarios. The bias expression in (2) shows that, as expected, the amount of bias in the hierarchical model depends on how well the perceived detectability captures the actual likelihood of detecting/missing the species at occupied sites.

In summary, based on our simulations and theoretical results, we can conclude that the hierarchical model is also less biased than the naïve model when detectability varies across sites, for instance as a result of variation in abundance. We also note that there are extensions of the hierarchical occupancy model that explicitly allow heterogeneous detection probabilities [21], [32]. These should be considered when modelling variation in detection probability as a function of environmental variables is not possible and it is likely that one of the assumptions of the basic model has been violated.

## Discussion

WLD present an overly negative picture of the performance of hierarchical occupancy models, questioning their value to the extent of suggesting that in general “ignoring non-detection can actually be better than trying to adjust for it”. Disregarding detectability implies modelling ‘where the species is *detected’* rather than ‘where the species *occurs’*, a quantity that can also be derived from the hierarchical model estimates. WLD implicitly suggest resorting to this alternative metric, but we expect that focusing on a metric that represents a mix of biological and sampling processes will not be satisfactory for most applications. For instance, [10] illustrate how disregarding imperfect detection can crucially compromise the identification of optimal habitat for a species, and hence misguide any spatial prioritization based on those results. When the target is to estimate occupancy probabilities, ignoring detectability is a problem even if it is constant. Ignoring imperfect detection is even more problematic when detectability is a function of covariates, as occupancy trends (spatial or temporal) can then also be biased. When wishing to reliably infer where a species *is* (instead of where is likely to be *observed*), then the necessary steps should be taken during design, collection and analysis of the data to minimize the effects of the sampling process (e.g., detectability).

WLD claim that “the extra data collection and modelling effort to try to adjust for non-detection is simply not worthwhile”. However, modelling detectability is not as hard as WLD would have readers believe. One does not necessarily need more sampling effort; instead the data need simply be collected and recorded in a way that is informative about the detection process [10]. Furthermore, the analysis is facilitated by a range of freely available software tools developed for hierarchical occupancy model fitting, comparison and prediction [27], [30], [33], [34]. As in WLD, we used maximum-likelihood for inference, but note that hierarchical occupancy models can also be easily fitted in the Bayesian framework using free tools such as WinBUGs/OpenBUGS [35] or JAGS [36]; for examples of code see [37]. Given WLD's and our difficulties in obtaining reliable parameter estimates with VGAM, we tentatively caution against its use for these applications, particularly with few repeat surveys.

WLD partly support their argument by pointing out that hierarchical occupancy models can produce estimates that are imprecise or at the boundary of the parameter space and that they can have problems with multiple solutions when the sample size is small. We have shown why we believe that WLD have overstated the severity of these issues. It is undeniable that, as with any type of statistical model, estimator performance will degrade as the sample size decreases, but in itself this does not justify discarding a method (this would suggest abandoning all statistical inference). Sample sizes can be too small to robustly infer species occupancy but disregarding detectability does not solve this situation.

We believe that accounting for detectability is important as otherwise it is impossible to know whether the “occupancy” estimates (even if precise) are accurate or not. In the naïve model, the occupancy and detection processes are confounded. One can find examples where disregarding detectability leads to estimators with better properties (in terms of MSE) but, since the same data can be produced by very different occupancy-detection scenarios, as shown in our simulations, we can never be confident that the naïve estimates reflect true occupancy unless detection is known to be perfect.

In contrast, the hierarchical occupancy model separates the occupancy and detection processes. If overall detection is nearly perfect (i.e., at occupied sites the probability of at least one detection for *K* repeat surveys, 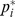, is practically 1), this partition is not detrimental: we will simply obtain the same estimates as in the naïve model. The benefit comes when overall detection is imperfect; then the estimates of occupancy 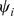 and naïve occupancy 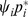 differ. WLD are concerned with the fact that the occupancy estimates can be imprecise. However, this imprecision should not be interpreted as a failure of the model, but rather a problem of insufficient data; it honestly represents the uncertainty. The model is indicating that there are alternative ways to explain the observed detections that involve very different occupancy probabilities. It is telling us all that can be reliably said about species occupancy with the available data. Being realistic about the uncertainty in estimates is fundamentally important, especially where estimates are to be used in any form of decision-making. Where estimates are too uncertain, the take-home message should be that more data may be required to make robust inference about the system in question, rather than turning to (naïve) estimators that can be arbitrarily biased.

WLD's key result is that hierarchical occupancy models do not perform any better than the naïve model when detectability depends on abundance, regardless of the amount of survey data, and that this “undermines the rationale for occupancy modelling”. We have shown that their result arises from a particular choice and limited interpretation of a specific scenario, which involves occupied sites in which the species is virtually undetectable while detectability is relatively large for other sites. We have demonstrated how the hierarchical model clearly outperforms the naïve model in other scenarios where heterogeneity is still substantial. We also show how this difference in performance is more apparent as the number of sites increases even if some bias remains when the number of sites is large. The basic hierarchical model is asymptotically biased when there is unaccounted heterogeneity in detection, as already pointed out by [21]. However, the fact that breaking a model assumption (no heterogeneity in *p*) may induce bias does not justify violating an additional assumption (perfect detection). The aim should instead be to achieve better estimation, minimizing the effect of these issues during design, data collection and analysis, for instance by considering model extensions that explicitly account for heterogeneous detection probabilities [21], [32]. This issue links with the problem of identifiability in mixture models for heterogeneous detectability raised by [38] in the context of capture/recapture abundance estimation methods, and which [21] re-examines for occupancy models. Alternative detection structures can fit the data equally well while providing different abundance or occupancy estimates. However, this problem is greatly reduced when the mass of the distribution describing detectability is moved away from zero [21]. This suggests that for reliable inference, our sampling methods must ensure non-negligible chances of detection when the species is present [1], [39].

In conclusion, although we fully agree with WLD about the need to be honest about the limitations of statistical procedures, we do not share their opinion that accounting for detectability is “very difficult” in general and that it is better to disregard the fact that detection can, and usually will, be imperfect. The difficulty is not so much in the modelling of detectability, but in imperfect detection itself. We do not claim that the modelling stage is straightforward. Indeed coming up with useful models for real data can be highly challenging. There will be cases for which meaningful parameter estimates cannot be obtained with the available data regardless of one's statistical skills. Unfortunately, and as much as one may desire it, naïve estimates are not a solution to this problem.

## Supporting Information

Appendix S1 **Full simulation results (scenarios A–B).**
(PDF)

Appendix S2 **Additional simulation results (no heterogeneity): MSE and coverage for constant models.**
(PDF)

Appendix S3 **Theoretical results for the abundance scenarios (B).**
(PDF)

Appendix S4 **Computer code (core simulation functions).**
(ZIP)
