# Supplementary material for: Ignoring Imperfect Detection in Biological Surveys Is Dangerous: A Response to ‘Fitting and Interpreting Occupancy Models'
Source: PLoS One. 2014 Jul 30;9(7):e99571. doi: 10.1371/journal.pone.0099571 (PMC4116132; doi:10.1371/journal.pone.0099571)
Supplement: Appendix S1 — Full simulation results (scenarios A–B). (PDF) [file pone.0099571.s001.pdf]

Appendix S1: Full simulation results (scenarios A-B)

This appendix contains the full simulations results for the simulated scenarios (table S1.1). For all scenarios we tested all the combinations of the following sampling sizes: $S = 55, 110$ or 165 sites and $K = 2, 3, 4$ or 5 replicate surveys per site

Table S1.1 Simulated scenarios

	Occupancy probability	Detection probability
Scenario A1*	$\psi = 0.4$	$\text{logit}(p_i) = -0.533 + 0.22x_i$
Scenario A2	$\psi = 0.8$	$\text{logit}(p_i) = -2.0 + 0.20x_i$
Scenario B1*	$\psi = 0.4$	$p_i \sim \text{Beta}(0.5,1)$ for $x_i \in \{1, 2\}$,<br>$p_i \sim \text{Beta}(1,1)$ for $x_i = 3$,<br>$p_i \sim \text{Beta}(10,2)$ for $x_i \in \{4, 5\}$.
Scenario B2	$\psi = 0.4$	$p_i \sim \text{Beta}(3,6)$ for $x_i \in \{1, 2\}$,<br>$p_i \sim \text{Beta}(5,5)$ for $x_i = 3$,<br>$p_i \sim \text{Beta}(10,2)$ for $x_i \in \{4, 5\}$.
Scenario B3	$\psi = 0.8$	$p_i \sim \text{Beta}(3,6)$ for $x_i \in \{1, 2\}$,<br>$p_i \sim \text{Beta}(5,5)$ for $x_i = 3$,<br>$p_i \sim \text{Beta}(10,2)$ for $x_i \in \{4, 5\}$.

* Scenario explored by WLD.

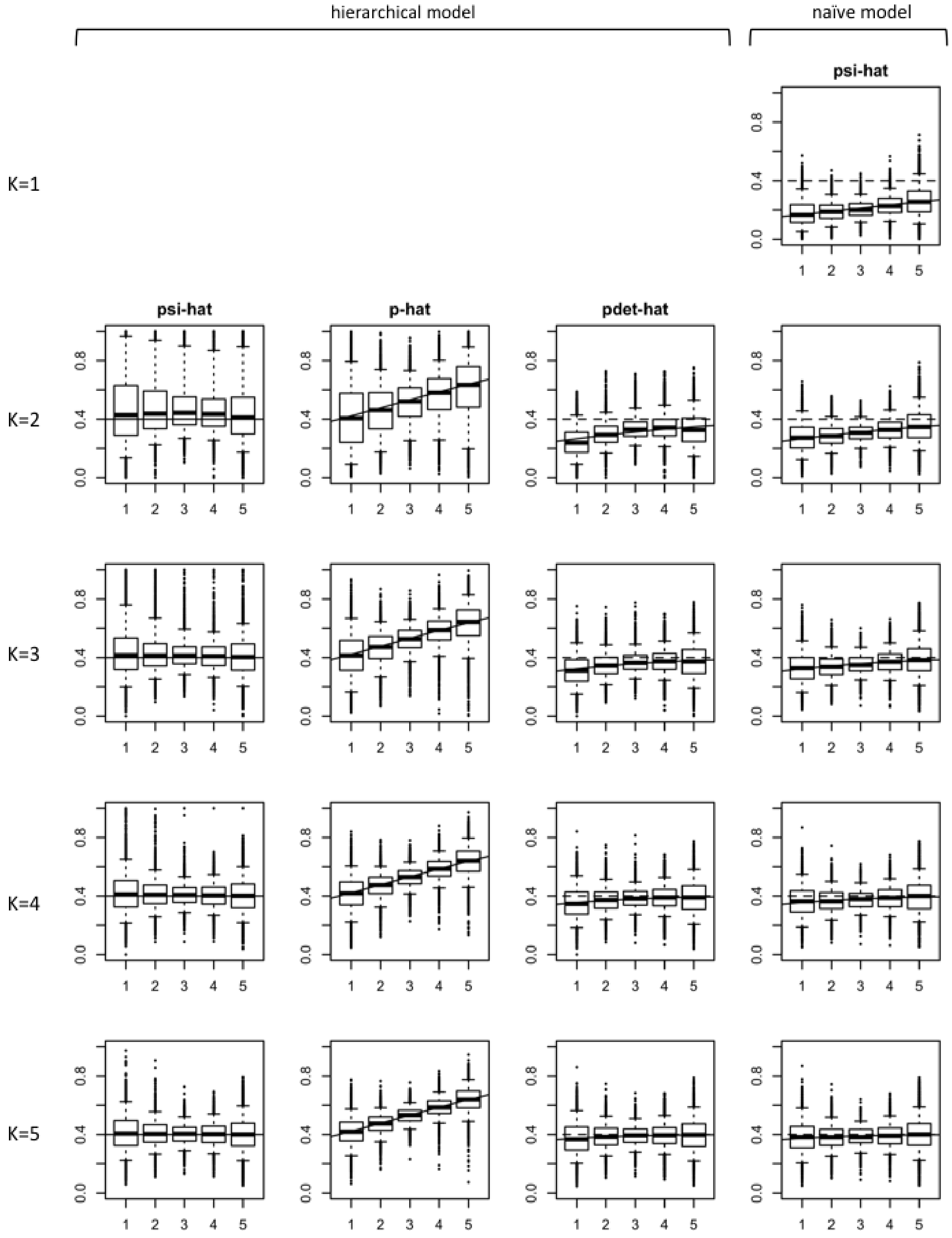

Figure S1.1: Scenario A1, $S = 55$

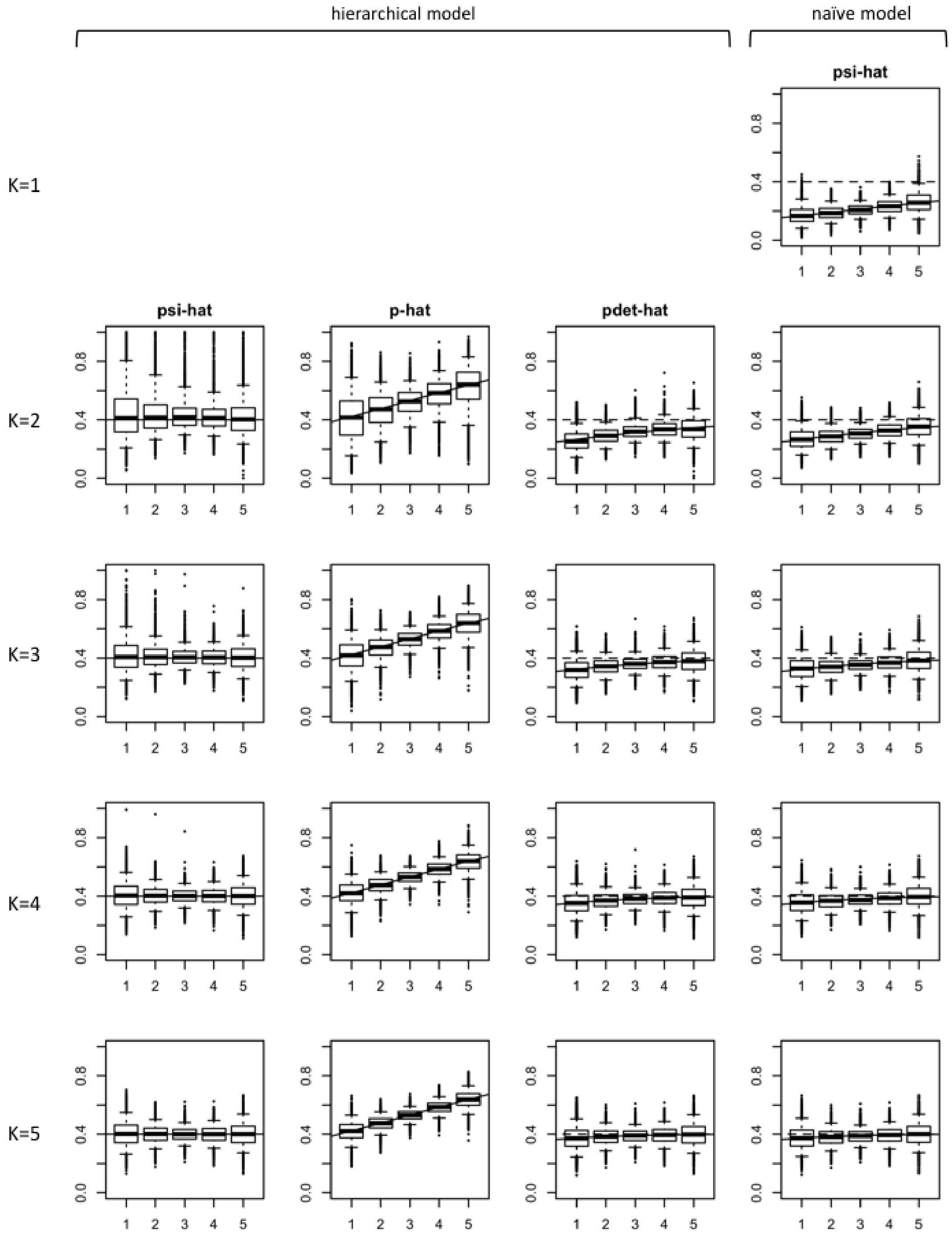

Figure S1.2: Scenario A1, $S = 110$

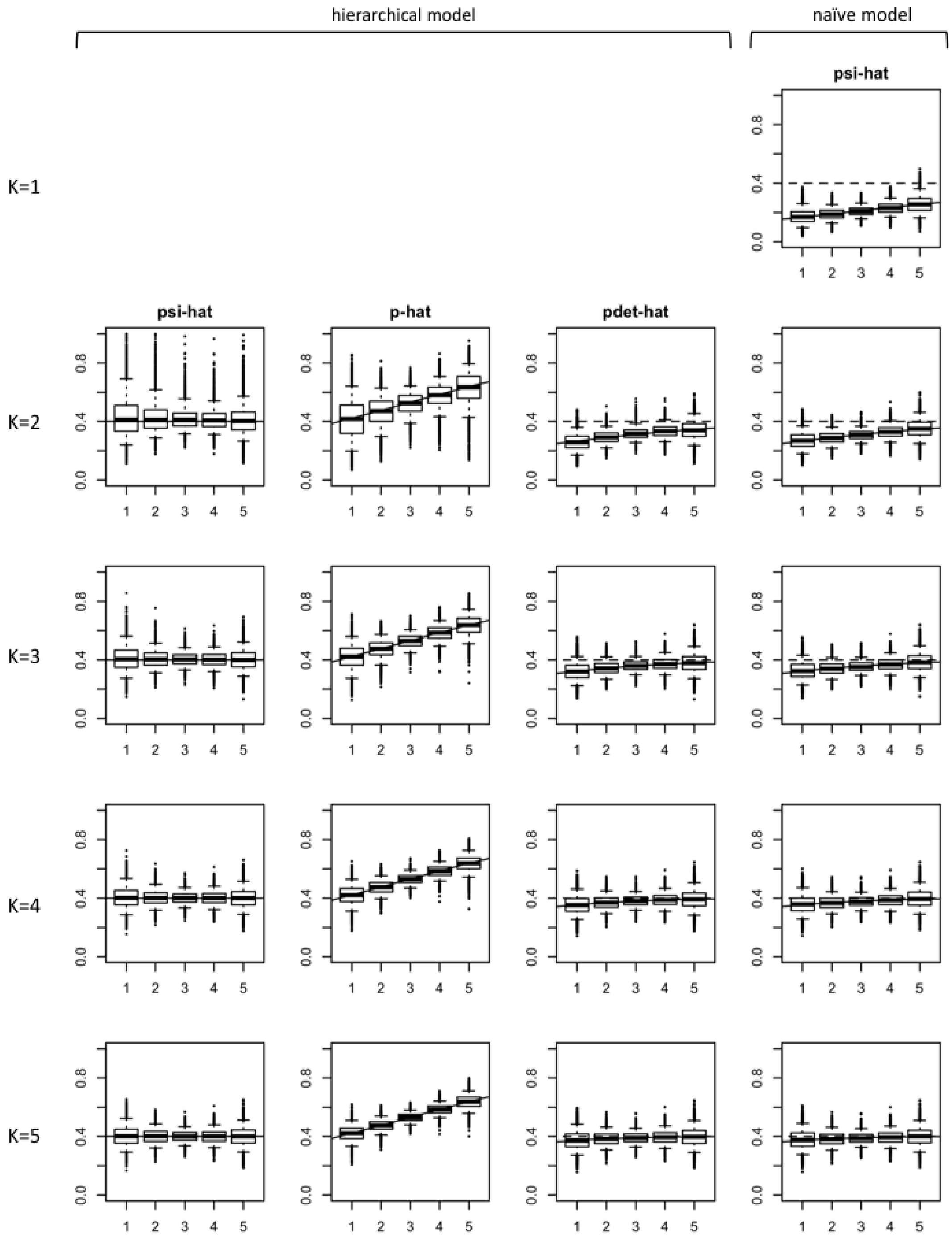

Figure S1.3: Scenario A1, $S = 165$

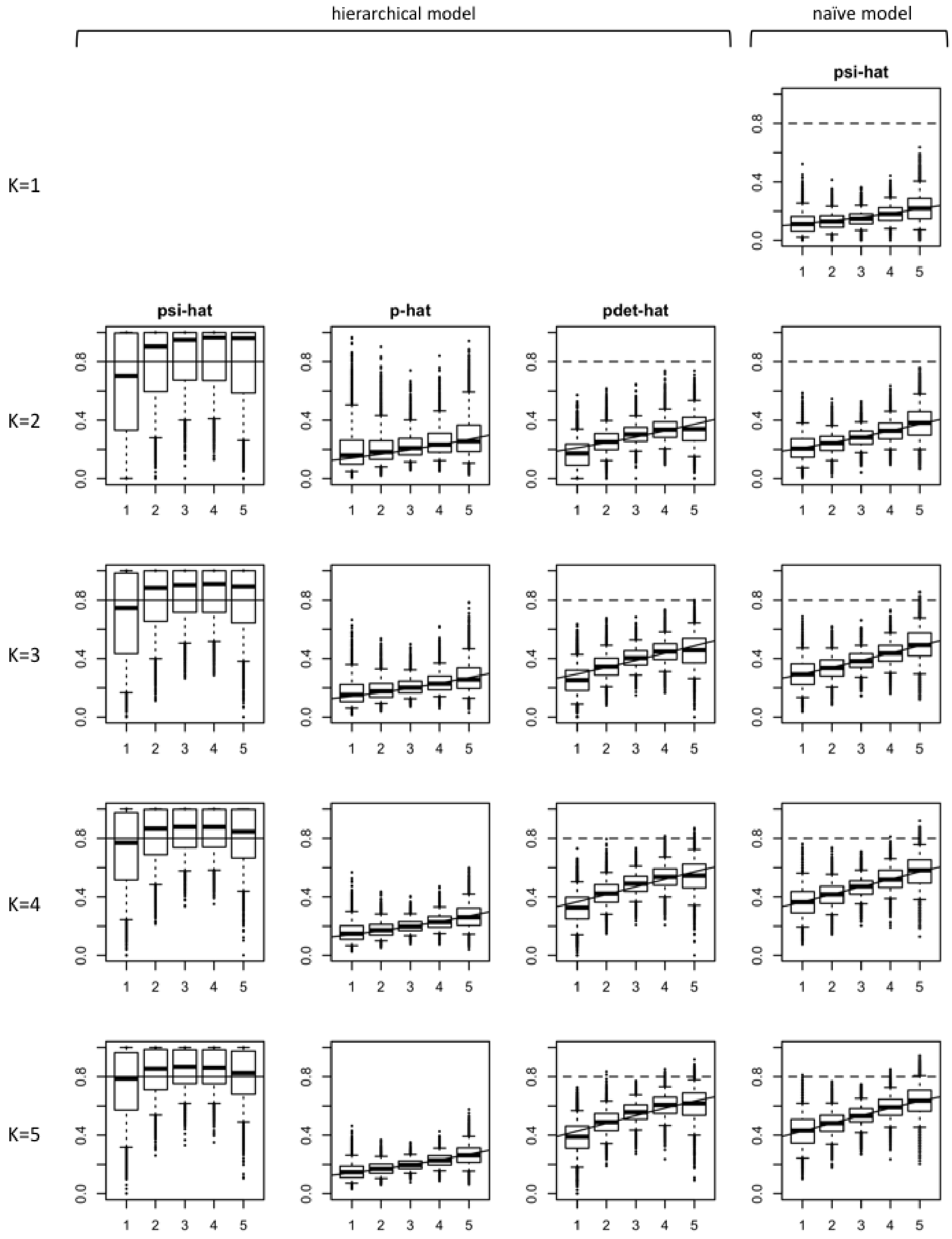

Figure S1.4: Scenario A2, $S = 55$

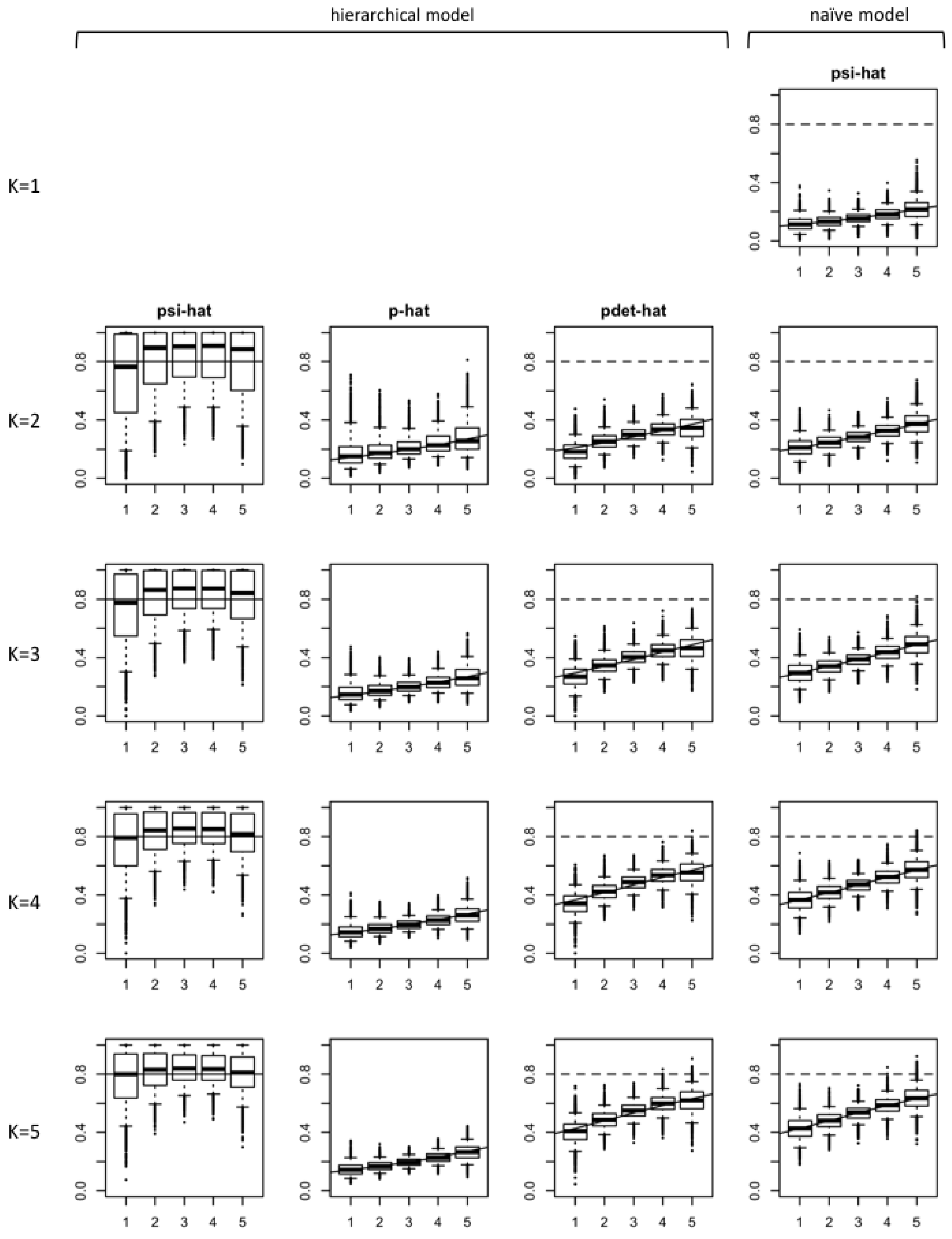

Figure S1.5: Scenario A2, $S = 110$

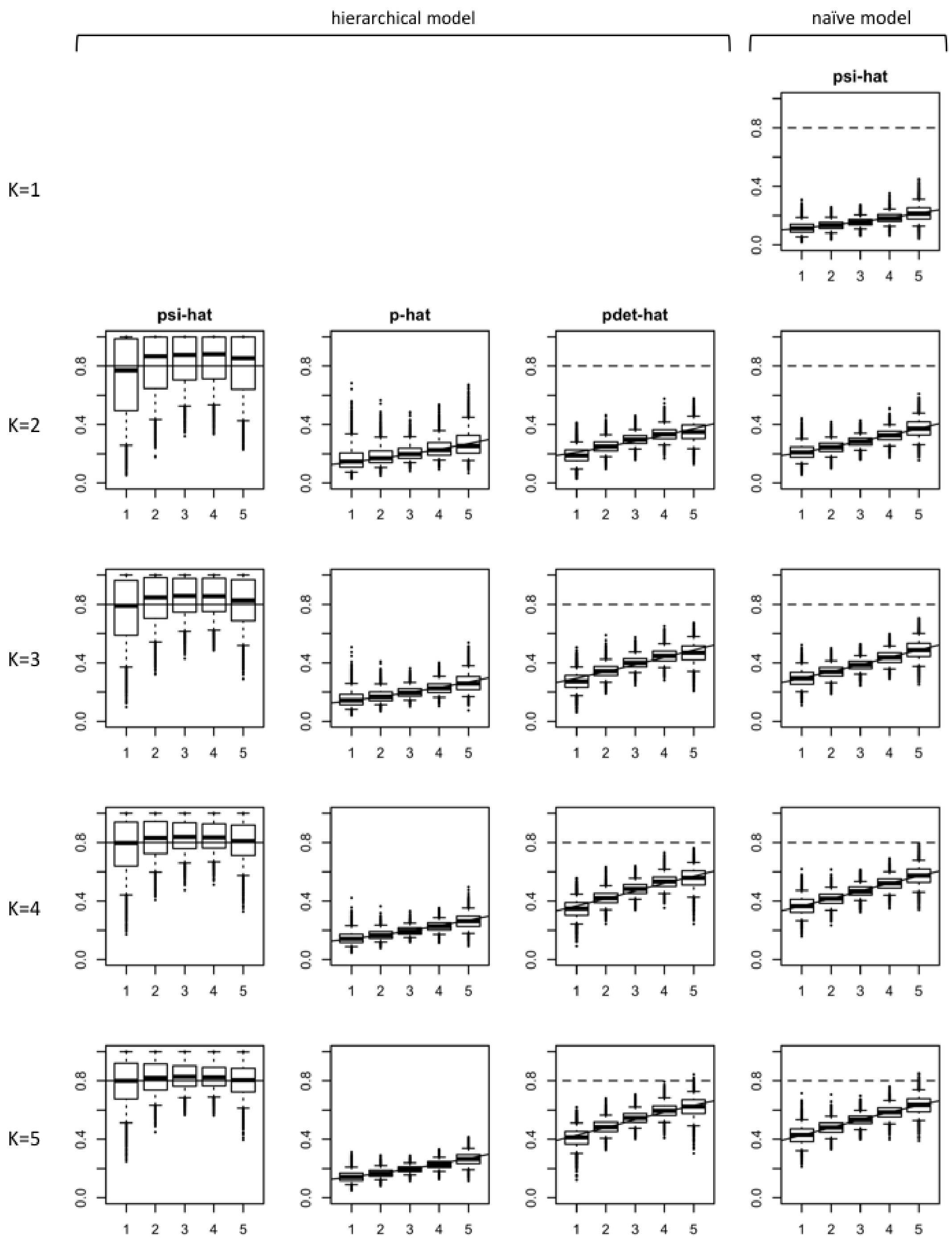

Figure S1.6: Scenario A2, $S = 165$

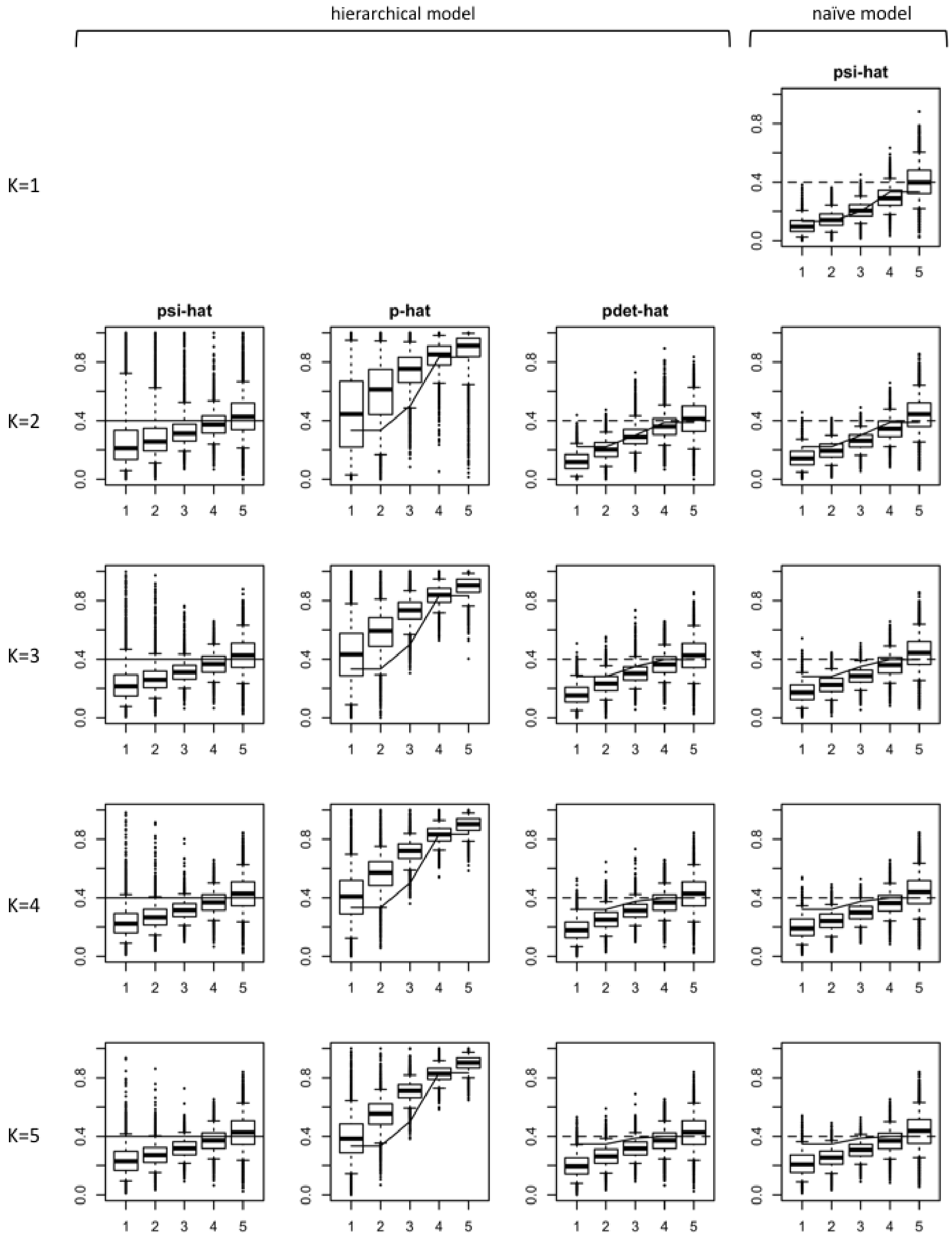

Figure S1.7: Scenario B1, $S = 55$

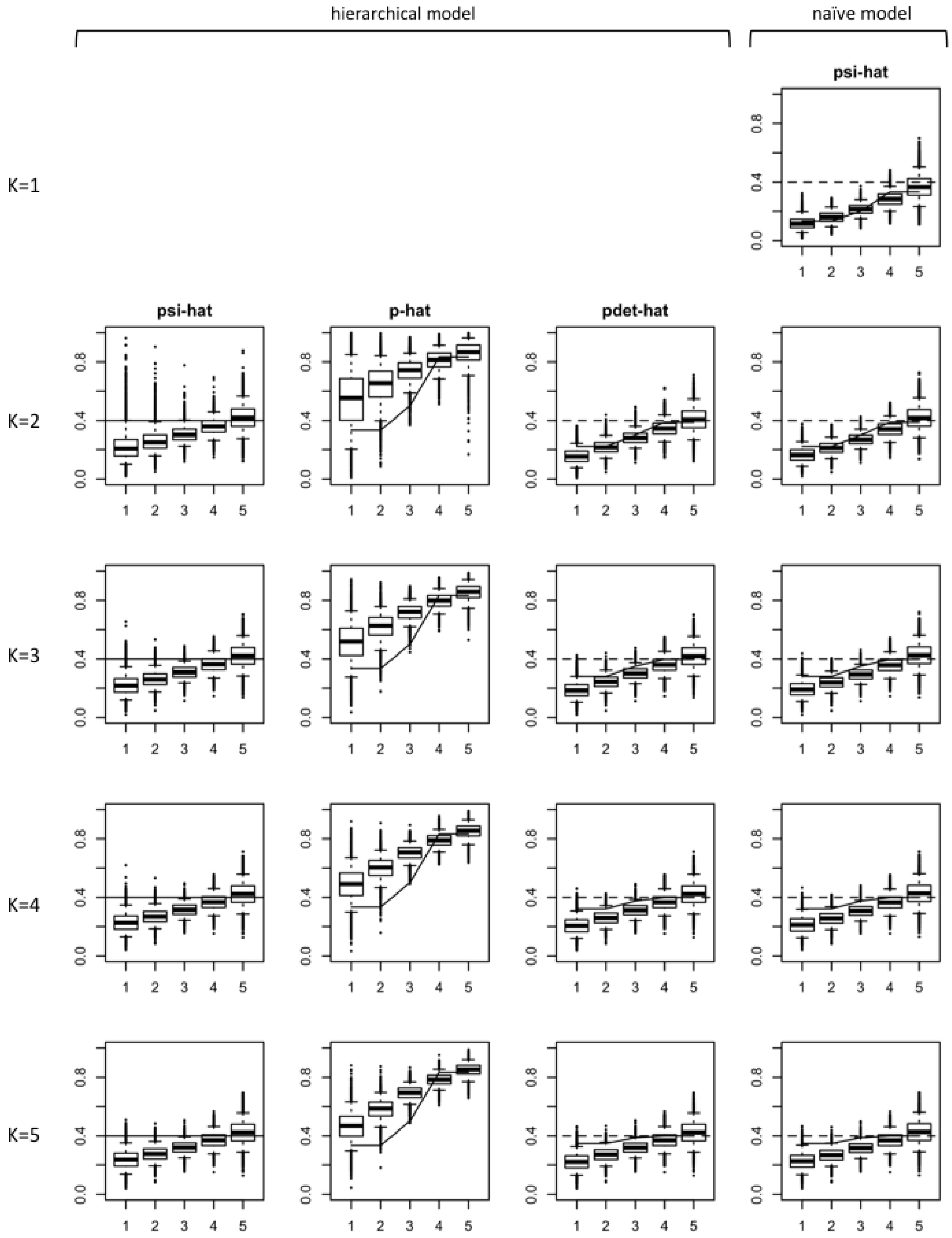

Figure S1.8: Scenario B1, $S = 110$

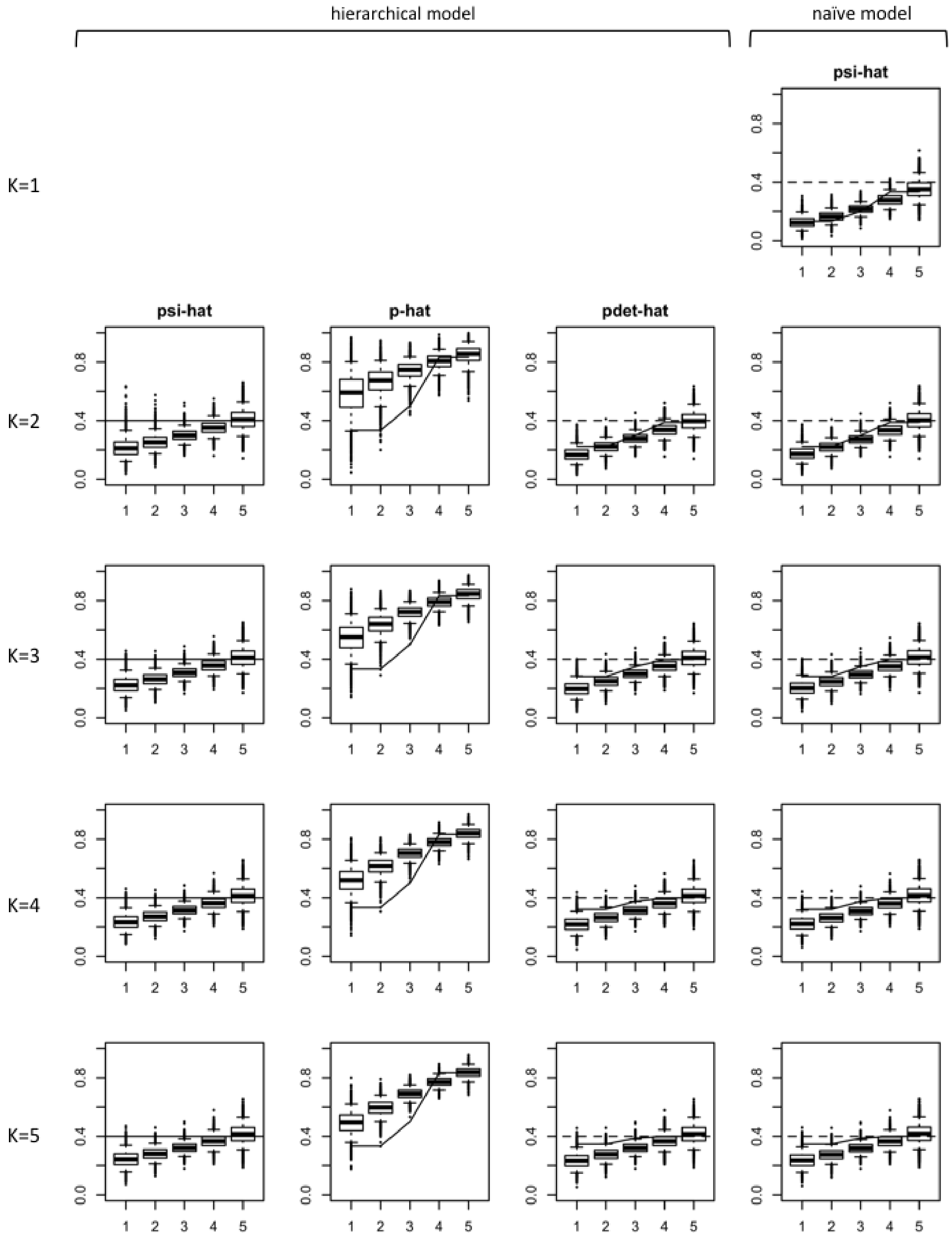

Figure S1.9: Scenario B1, $S = 165$

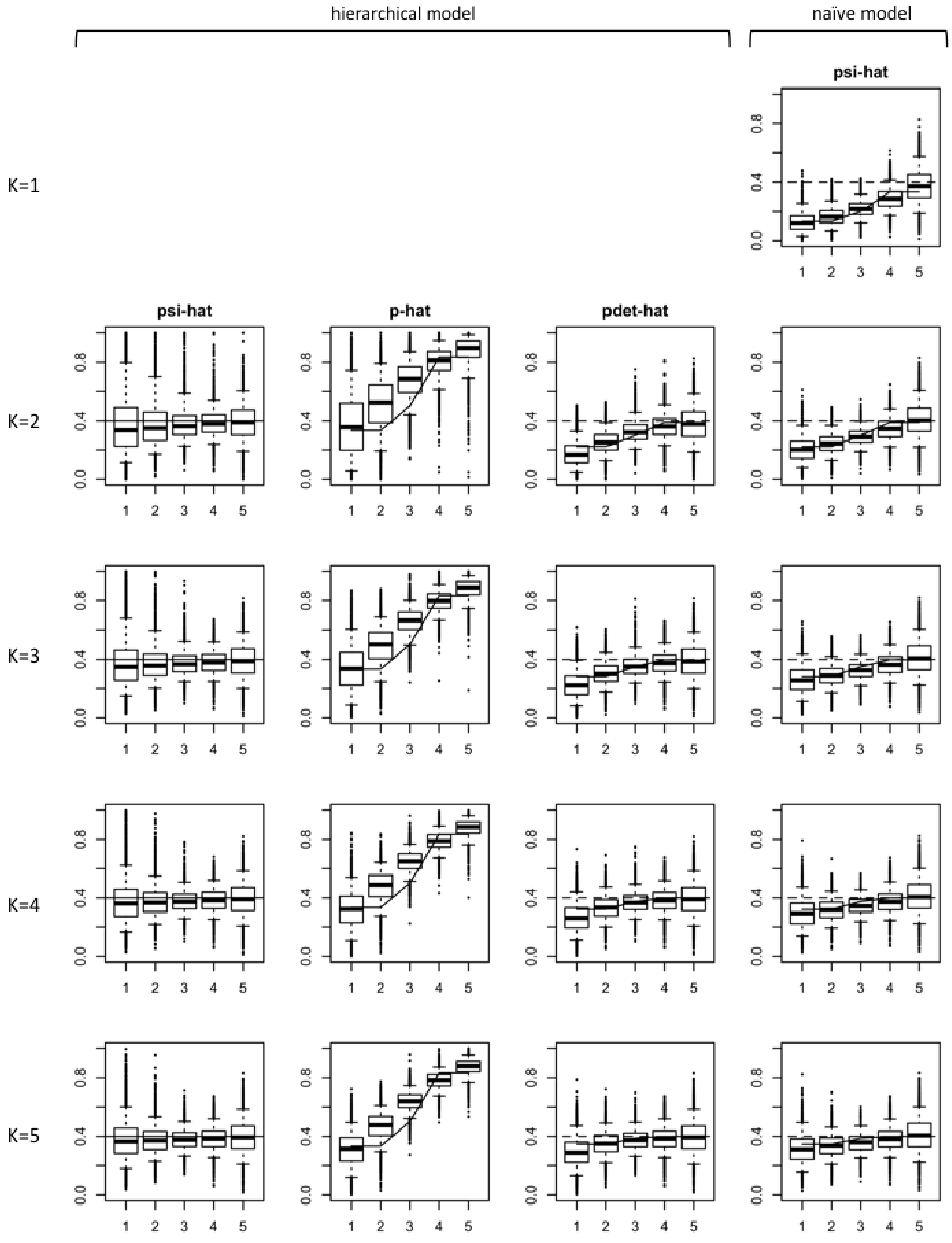

Figure S1.10: Scenario B2, $S = 55$

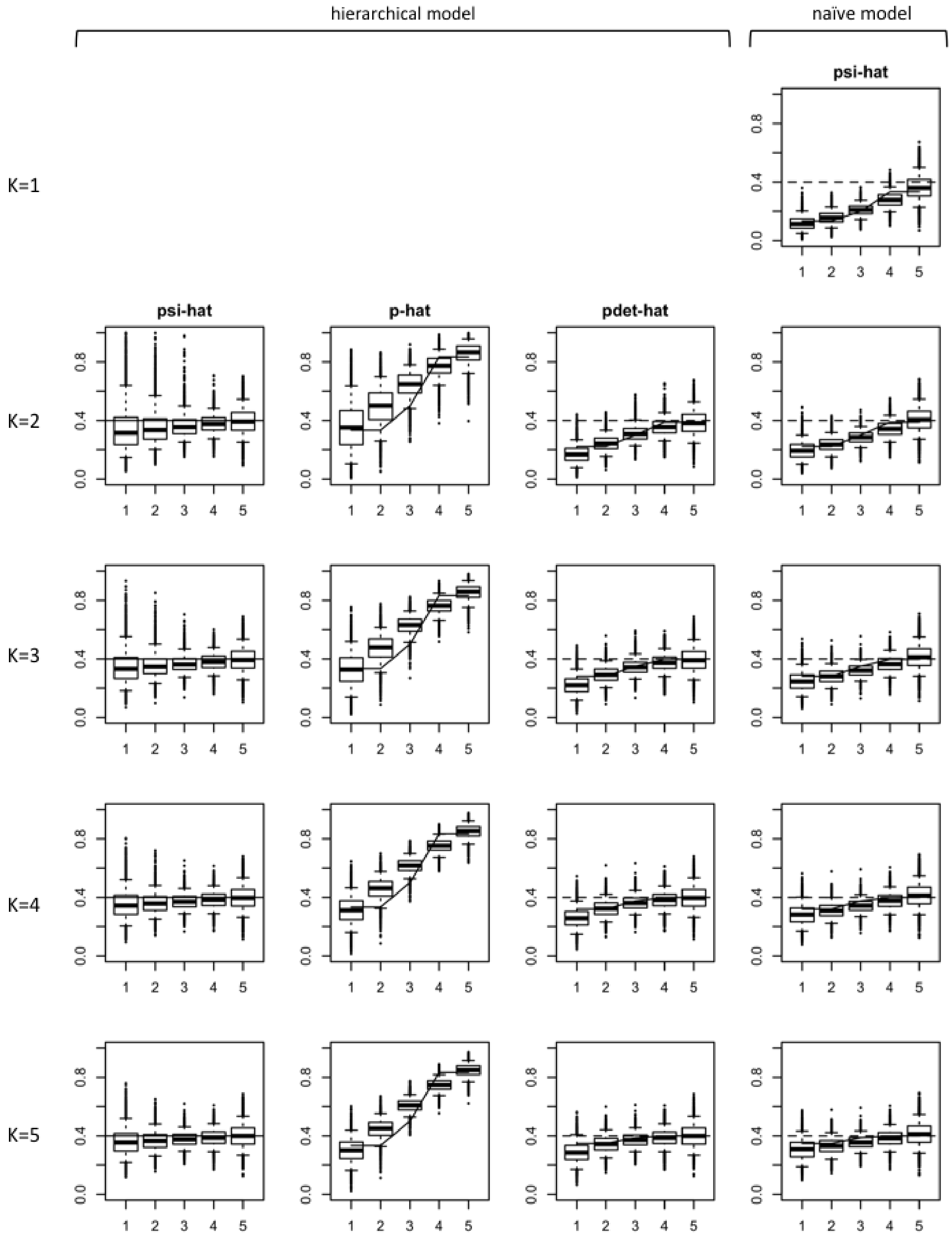

Figure S1.11: Scenario B2, $S = 110$

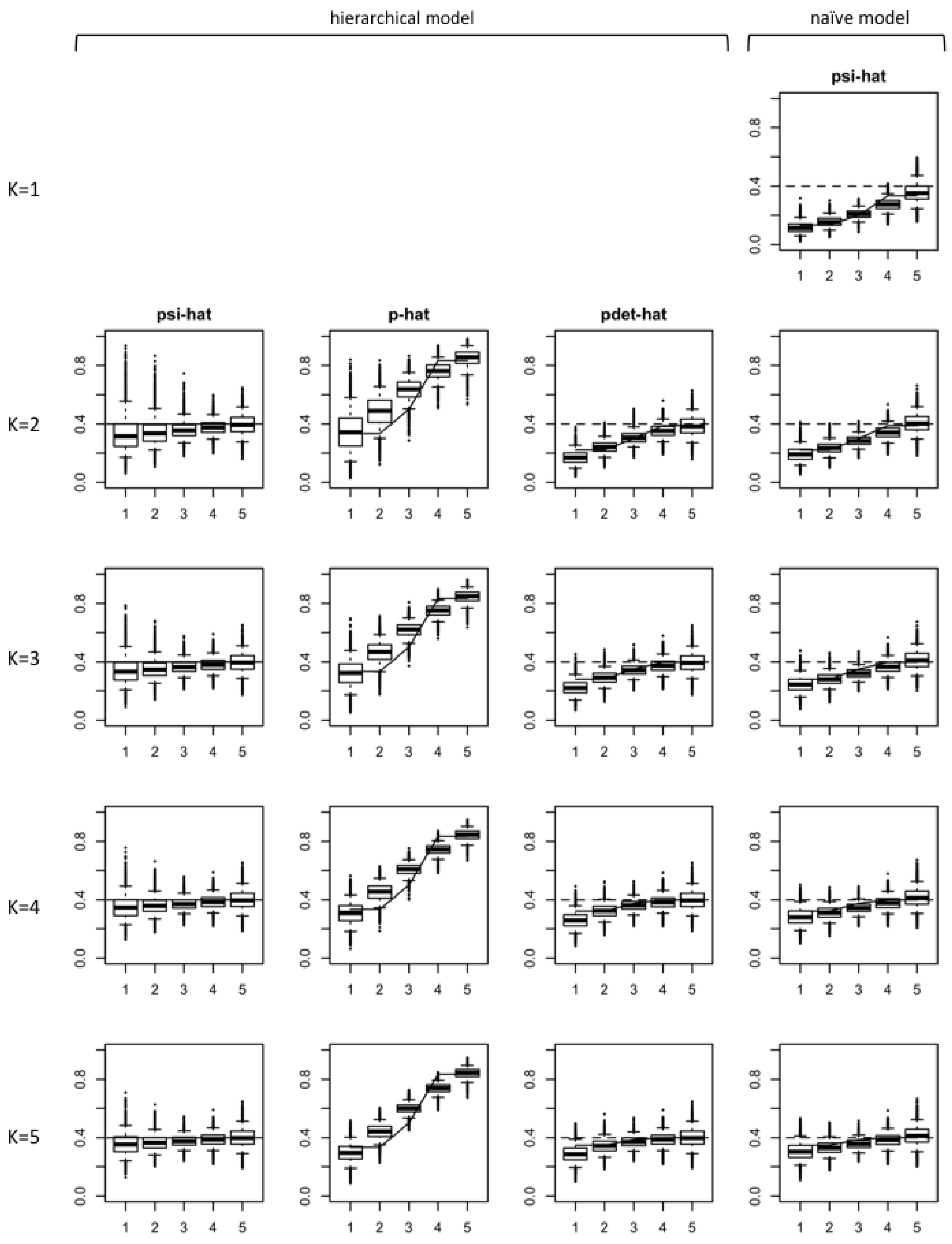

Figure S1.12: Scenario B2, $S = 165$

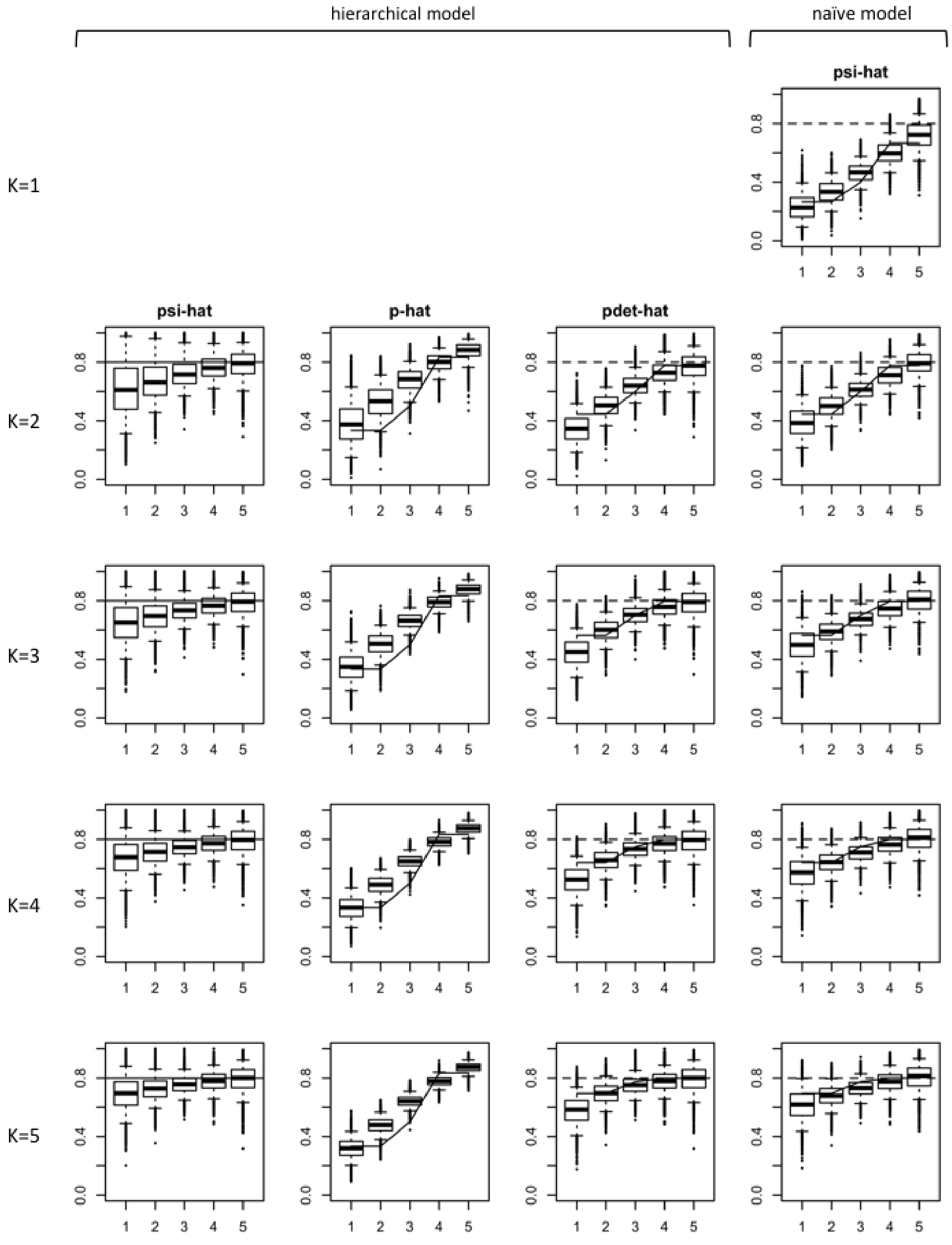

Figure S1.13: Scenario B3, $S = 55$

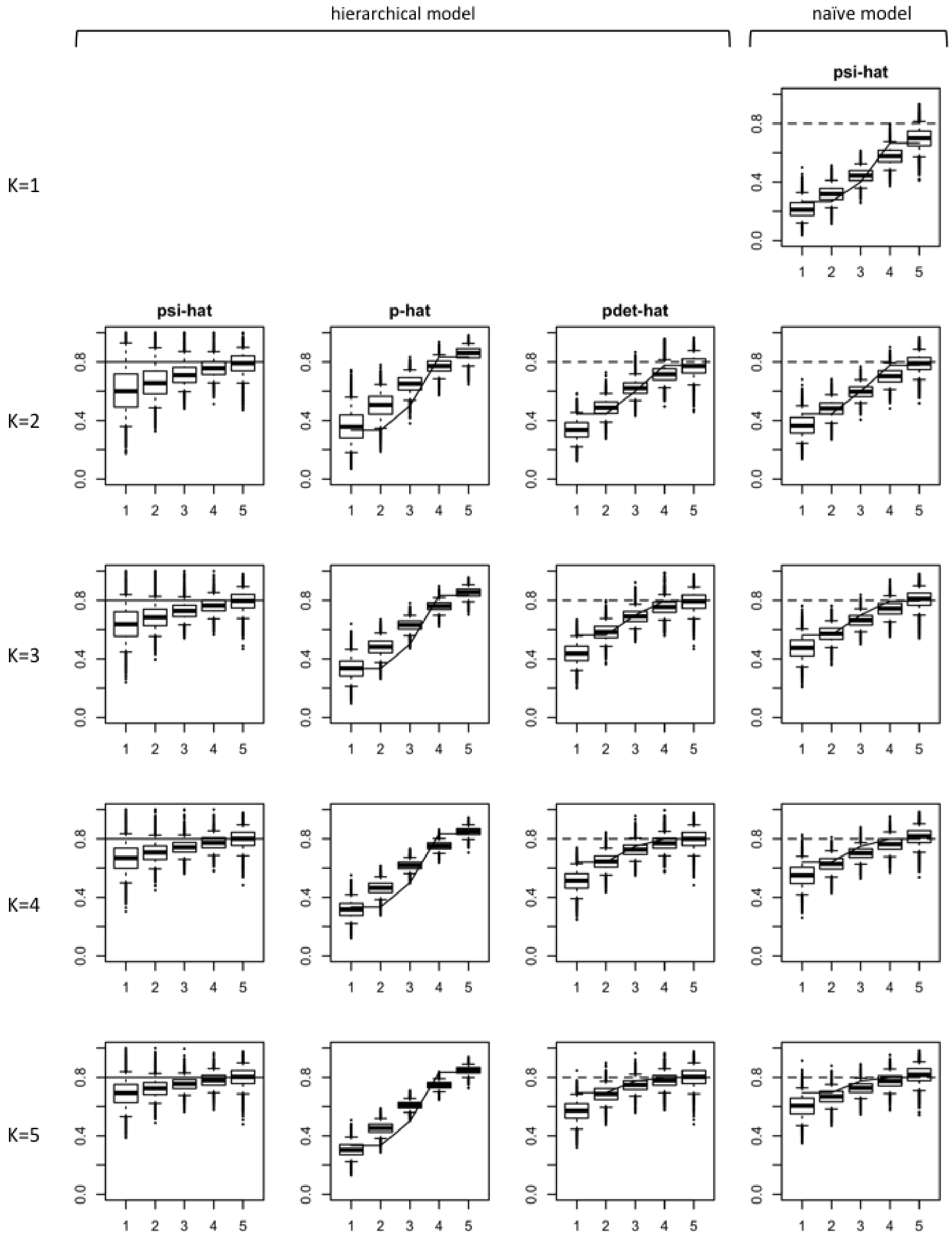

Figure S1.14: Scenario B3, $S = 110$

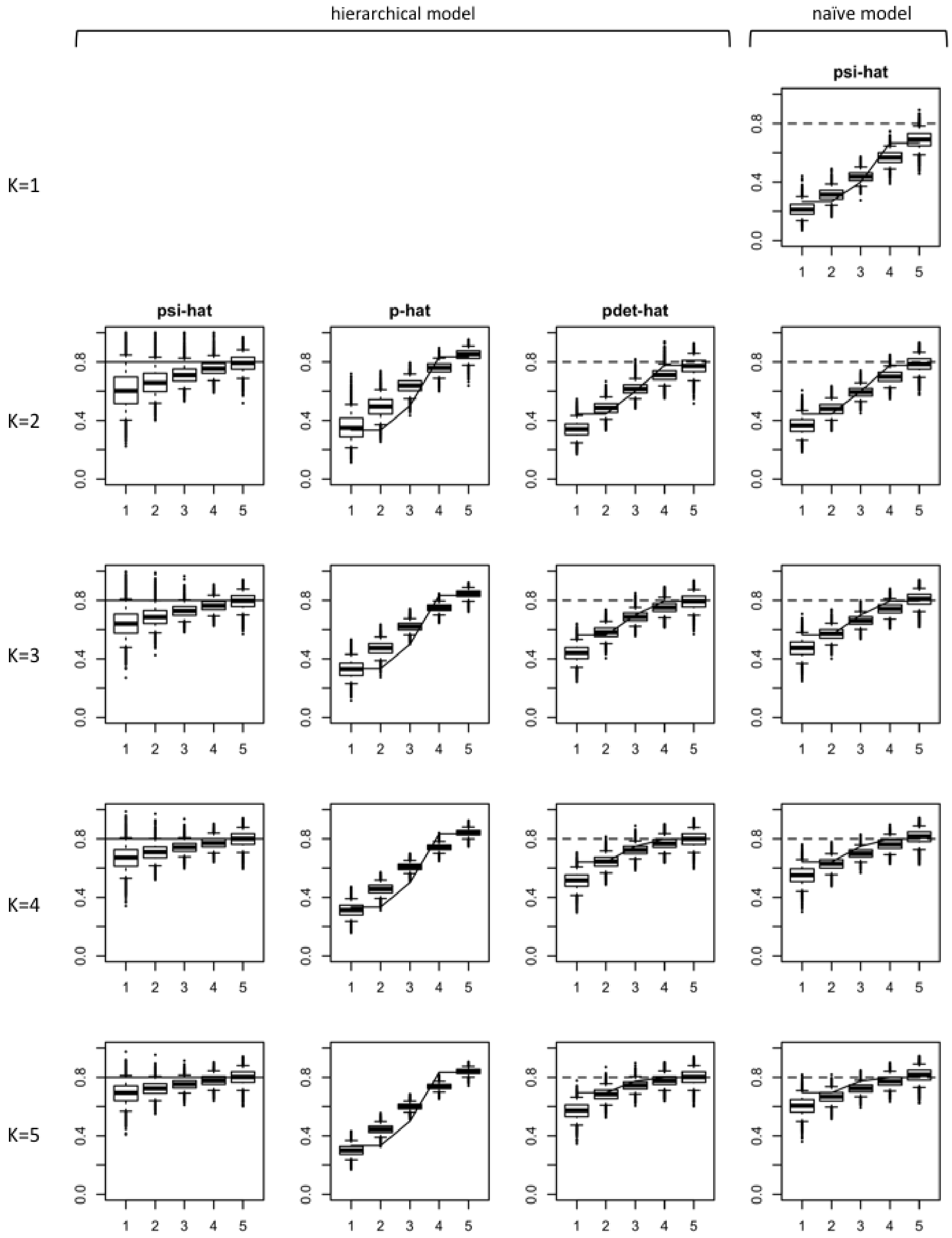

Figure S1.15: Scenario B3, $S = 165$
