# Supplementary material for: Ignoring Imperfect Detection in Biological Surveys Is Dangerous: A Response to ‘Fitting and Interpreting Occupancy Models'
Source: PLoS One. 2014 Jul 30;9(7):e99571. doi: 10.1371/journal.pone.0099571 (PMC4116132; doi:10.1371/journal.pone.0099571)
Supplement: Appendix S2 — Additional simulation results (no heterogeneity): MSE and coverage for constant models. (PDF) [file pone.0099571.s002.pdf]

Appendix S2: Additional simulation results (no heterogeneity): MSE and coverage for constant models.

This appendix contains the results of additional simulations that support our claims (in particular message 3). In these simulations we explored the parameter space by considering all combinations of ψ in $\{0.2,0.4,0.6,0.8\}$ and p in $\{0.1,0.3,0.5,0.7,0.9\}$. Both parameters were assumed constant within each simulation. We considered different sample sizes with all combinations of S in $\{55,110,165\}$ and K in $\{2,3,4,5\}$. Hence, in total we explored 240 scenarios (although for clarity we only report 180). We ran 5000 simulations for each scenario, analyzed the data with the naïve and hierarchical models (same methods as described for other simulations in the main text) and computed the mean square error (MSE) of the occupancy estimator for both models. We also computed the coverage of the confidence intervals for both models as the proportion of simulations in which the confidence interval for the occupancy estimate included the true occupancy value. Since asymptotic confidence intervals can behave poorly when sample size is small, for the naïve model we used Wilson's confidence interval, which is known to have good coverage properties [1], and for the hierarchical model we computed profile likelihood confidence intervals [2].

These simulations confirm that, although in some scenarios the naïve model can have better MSE than the hierarchical model (Table S2.1), this only happens for small sample sizes (low S and low K) and when occupancy and detection probabilities are small. As pointed out in the main text, the key problem is that the naïve model can produce the same apparently precise estimates for different scenarios including those where the estimator is very biased, i.e. it can be overconfident about results that can be very wrong. Our simulations confirm the poor coverage of the naïve model estimator (Table S2.2) when detection is imperfect. For instance, in only 0.2% of the simulations the naïve model's confidence interval included the true occupancy value when S = 110, $K = 2$, ψ = 0.8, p = 0.5. This problem is greater the lower the detection probability, the greater the occupancy probability and the greater the number of sampling sites. The naïve estimator is not a consistent estimator when detectability is imperfect (i.e., as the number of sampling sites increases, its MSE does not approach zero). As expected, in the hierarchical model the coverage is close to the nominal value.

In summary, when detection is imperfect and data are scarce, the hierarchical model can be imprecise and hence have a poorer MSE. However, this imprecision reflects the actual uncertainty in the data and the reported confidence interval tends to include the true parameter value. The naive model on the other hand can be overconfident about very wrong results.

Table S2.1 Mean square error (MSE) for naïve and hierarchical occupancy models

(a) naive

		K = 2					K = 3					K = 4				
	p	0.1	0.3	0.5	0.7	0.9	0.1	0.3	0.5	0.7	0.9	0.1	0.3	0.5	0.7	0.9
**S = 55**	psi 0.2	0.025	0.011	0.005	0.003	0.003	0.021	0.007	0.003	0.003	0.003	0.018	0.005	0.003	0.003	0.003
	0.4	0.104	0.042	0.014	0.005	0.004	0.088	0.022	0.007	0.005	0.004	0.070	0.014	0.004	0.004	0.005
	0.6	0.239	0.091	0.027	0.007	0.005	0.195	0.048	0.010	0.005	0.005	0.157	0.025	0.006	0.004	0.005
	0.8	0.423	0.159	0.045	0.009	0.003	0.343	0.079	0.014	0.004	0.003	0.278	0.041	0.006	0.003	0.003
**S = 110**	psi 0.2	0.027	0.011	0.004	0.002	0.001	0.021	0.006	0.002	0.002	0.001	0.018	0.004	0.002	0.001	0.001
	0.4	0.106	0.040	0.012	0.003	0.002	0.086	0.021	0.004	0.002	0.002	0.070	0.012	0.002	0.002	0.002
	0.6	0.239	0.088	0.025	0.005	0.002	0.193	0.043	0.008	0.002	0.002	0.157	0.022	0.004	0.002	0.002
	0.8	0.420	0.157	0.041	0.007	0.001	0.341	0.078	0.012	0.002	0.002	0.277	0.039	0.004	0.001	0.001
**S = 165**	psi 0.2	0.026	0.010	0.003	0.001	0.001	0.021	0.005	0.001	0.001	0.001	0.017	0.003	0.001	0.001	0.001
	0.4	0.106	0.039	0.011	0.003	0.002	0.086	0.020	0.004	0.002	0.001	0.071	0.010	0.002	0.001	0.002
	0.6	0.237	0.088	0.025	0.004	0.002	0.192	0.044	0.007	0.002	0.002	0.157	0.022	0.003	0.002	0.001
	0.8	0.420	0.155	0.042	0.007	0.001	0.344	0.076	0.011	0.002	0.001	0.277	0.038	0.004	0.001	0.001

(b) hierarchical

		K = 2					K = 3					K = 4				
	p	0.1	0.3	0.5	0.7	0.9	0.1	0.3	0.5	0.7	0.9	0.1	0.3	0.5	0.7	0.9
**S = 55**	psi 0.2	0.536	0.242	0.052	0.005	0.003	0.459	0.071	0.006	0.003	0.003	0.353	0.020	0.003	0.003	0.003
	0.4	0.289	0.088	0.023	0.006	0.004	0.213	0.031	0.007	0.005	0.004	0.152	0.013	0.005	0.004	0.005
	0.6	0.147	0.057	0.020	0.007	0.005	0.104	0.027	0.008	0.005	0.005	0.081	0.016	0.005	0.004	0.005
	0.8	0.091	0.033	0.015	0.006	0.003	0.063	0.019	0.008	0.003	0.003	0.051	0.013	0.006	0.003	0.003
**S = 110**	psi 0.2	0.484	0.101	0.007	0.002	0.001	0.329	0.014	0.002	0.002	0.001	0.240	0.005	0.002	0.001	0.001
	0.4	0.243	0.052	0.008	0.003	0.002	0.139	0.013	0.003	0.002	0.002	0.081	0.005	0.002	0.002	0.002
	0.6	0.119	0.037	0.009	0.003	0.002	0.075	0.014	0.004	0.002	0.002	0.057	0.009	0.003	0.002	0.002
	0.8	0.077	0.024	0.009	0.003	0.001	0.049	0.015	0.004	0.002	0.002	0.034	0.020	0.003	0.001	0.002
**S = 165**	psi 0.2	0.446	0.048	0.004	0.001	0.001	0.256	0.008	0.001	0.001	0.001	0.138	0.002	0.001	0.001	0.001
	0.4	0.207	0.031	0.004	0.002	0.002	0.104	0.007	0.002	0.002	0.001	0.060	0.003	0.002	0.001	0.002
	0.6	0.098	0.028	0.006	0.002	0.002	0.062	0.008	0.002	0.002	0.002	0.045	0.007	0.002	0.002	0.001
	0.8	0.065	0.020	0.006	0.002	0.001	0.041	0.012	0.003	0.001	0.001	0.028	0.007	0.003	0.001	0.001

Note: Red indicates scenarios where the MSE in the naïve model is lower than in the hierarchical model by 10% or more; green is used for the opposite (i.e. MSE_h <=0.9 MSE_n).

Table S2.2 Confidence interval coverage for naïve and hierarchical occupancy models

(a) naive

		K = 2					K = 3					K = 4				
		p					p					p				
	psi	0.1	0.3	0.5	0.7	0.9	0.1	0.3	0.5	0.7	0.9	0.1	0.3	0.5	0.7	0.9
**S = 55**	0.2	0.03	0.50	0.84	0.94	0.94	0.07	0.75	0.92	0.95	0.94	0.19	0.85	0.92	0.93	0.94
	0.4	0.00	0.13	0.72	0.94	0.97	0.00	0.49	0.90	0.95	0.97	0.01	0.72	0.97	0.97	0.96
	0.6	0.00	0.00	0.42	0.91	0.95	0.00	0.13	0.83	0.95	0.96	0.00	0.45	0.94	0.95	0.96
	0.8	0.00	0.00	0.06	0.70	0.93	0.00	0.00	0.52	0.91	0.93	0.00	0.07	0.81	0.93	0.94
**S = 110**	0.2	0.00	0.23	0.79	0.94	0.97	0.01	0.58	0.93	0.95	0.96	0.02	0.80	0.95	0.96	0.95
	0.4	0.00	0.01	0.45	0.91	0.95	0.00	0.15	0.85	0.96	0.95	0.00	0.45	0.95	0.95	0.96
	0.6	0.00	0.00	0.12	0.82	0.96	0.00	0.01	0.69	0.96	0.97	0.00	0.16	0.88	0.95	0.96
	0.8	0.00	0.00	0.00	0.53	0.96	0.00	0.00	0.31	0.91	0.95	0.00	0.01	0.78	0.96	0.96
**S = 165**	0.2	0.00	0.08	0.66	0.94	0.97	0.00	0.43	0.91	0.96	0.95	0.00	0.72	0.95	0.95	0.96
	0.4	0.00	0.00	0.24	0.86	0.94	0.00	0.04	0.74	0.95	0.96	0.00	0.28	0.91	0.95	0.96
	0.6	0.00	0.00	0.03	0.74	0.95	0.00	0.00	0.48	0.94	0.95	0.00	0.05	0.85	0.95	0.96
	0.8	0.00	0.00	0.00	0.41	0.94	0.00	0.00	0.16	0.89	0.95	0.00	0.00	0.66	0.96	0.97

(b) hierarchical

		K = 2					K = 3					K = 4				
		p					p					p				
	psi	0.1	0.3	0.5	0.7	0.9	0.1	0.3	0.5	0.7	0.9	0.1	0.3	0.5	0.7	0.9
**S = 55**	0.2	0.88	0.96	0.95	0.95	0.95	0.94	0.95	0.96	0.96	0.96	0.94	0.94	0.94	0.94	0.96
	0.4	0.96	0.95	0.95	0.96	0.96	0.97	0.94	0.95	0.94	0.96	0.98	0.94	0.96	0.96	0.94
	0.6	0.97	0.97	0.95	0.95	0.93	0.98	0.95	0.96	0.94	0.93	0.98	0.95	0.95	0.94	0.94
	0.8	0.98	0.98	0.96	0.94	0.95	0.99	0.98	0.94	0.95	0.96	0.98	0.95	0.95	0.95	0.96
**S = 110**	0.2	0.95	0.95	0.95	0.95	0.96	0.97	0.95	0.95	0.94	0.95	0.95	0.94	0.95	0.96	0.94
	0.4	0.98	0.94	0.95	0.95	0.94	0.98	0.95	0.94	0.95	0.93	0.96	0.96	0.96	0.94	0.95
	0.6	0.98	0.95	0.96	0.95	0.95	0.98	0.95	0.95	0.95	0.95	0.97	0.95	0.95	0.94	0.96
	0.8	0.98	0.97	0.95	0.95	0.95	0.98	0.95	0.96	0.95	0.95	0.98	0.96	0.94	0.96	0.96
**S = 165**	0.2	0.98	0.94	0.96	0.95	0.95	0.98	0.93	0.95	0.95	0.95	0.96	0.95	0.94	0.94	0.96
	0.4	0.98	0.96	0.96	0.95	0.94	0.97	0.95	0.95	0.95	0.96	0.96	0.95	0.95	0.94	0.96
	0.6	0.97	0.94	0.95	0.94	0.94	0.98	0.95	0.95	0.95	0.94	0.98	0.97	0.95	0.95	0.96
	0.8	0.98	0.96	0.95	0.94	0.93	0.98	0.96	0.94	0.94	0.93	0.98	0.96	0.95	0.94	0.96

Note: The white to green color scale reflects a gradient from poor to good coverage.

References:

1. Brown LD, Cai TT, DasGupta A, Agresti A, Coull BA, et al. (2001) Interval estimation for a binomial proportion. Statistical Science 16: 101-133.
2. Gimenez O, Choquet R, Lamor L, Scofield P, Fletcher D, et al. (2005) Efficient profile-likelihood confidence intervals for capture-recapture models. Journal of Agricultural Biological and Environmental Statistics 10: 184-196.
