# Supplementary material for: Ignoring Imperfect Detection in Biological Surveys Is Dangerous: A Response to ‘Fitting and Interpreting Occupancy Models'
Source: PLoS One. 2014 Jul 30;9(7):e99571. doi: 10.1371/journal.pone.0099571 (PMC4116132; doi:10.1371/journal.pone.0099571)
Supplement: Appendix S3 — Theoretical results for the abundance scenarios (B). (PDF) [file pone.0099571.s003.pdf]

Appendix S3: Theoretical results for the “abundance” scenarios (B)

This appendix contains the results of solving numerically the expected estimating equations for the “abundance” scenarios (B1-B3), which provide insight into the asymptotic bias of the models. The estimating equations can be found in the original paper (eqs 4-5 in WLD) as well as the calculations for the expectations of the data (in pp.12-13). These results corroborate our simulations and show how the hierarchical model outperforms the naïve model also when detectability is heterogeneous (only marginally in B1, but clearly in B2-B3). The asymptotic bias due to model misspecification (p assumed constant within each covariate category when it is heterogeneous) decreases as K increases.

Table S3.1 Solutions of the expected estimating equations

Scenario	Model	K	β_0	β_1	γ_0	γ_1
B1	$\psi(x_i), p(x_i)$	2	-1.576	0.244	-0.198	0.397
		5	-1.278	0.188	-0.693	0.475
		10	-1.049	0.141	-1.022	0.536
	$\psi(x_i), p = 1$	1	-2.365	0.356	--	--
		2	-1.880	0.302	--	--
		5	-1.344	0.203	--	--
		10	-1.058	0.144	--	--
B2	$\psi(x_i)p(x_i)$	2	-0.837	0.083	-1.281	0.621
		5	-0.604	0.039	-1.523	0.652
		10	-0.505	0.021	-1.639	0.669
	$\psi(x_i), p = 1$	1	-2.365	0.356	--	--
		2	-1.637	0.251	--	--
		5	-0.867	0.101	--	--
		10	-0.559	0.035	--	--
B3	$\psi(x_i)p(x_i)$	2	0.325	0.202	-1.269	0.616
		5	0.815	0.113	-1.508	0.648
		10	1.101	0.061	-1.637	0.668
	$\psi(x_i), p = 1$	1	-1.774	0.521	--	--
		2	-0.931	0.451	--	--
		5	0.287	0.239	--	--
		10	0.964	0.095	--	--

Note: $\text{logit}(\psi_i) = \beta_0 + \beta_1 x_i$ and $\text{logit}(p_i) = \gamma_0 + \gamma_1 x_i$

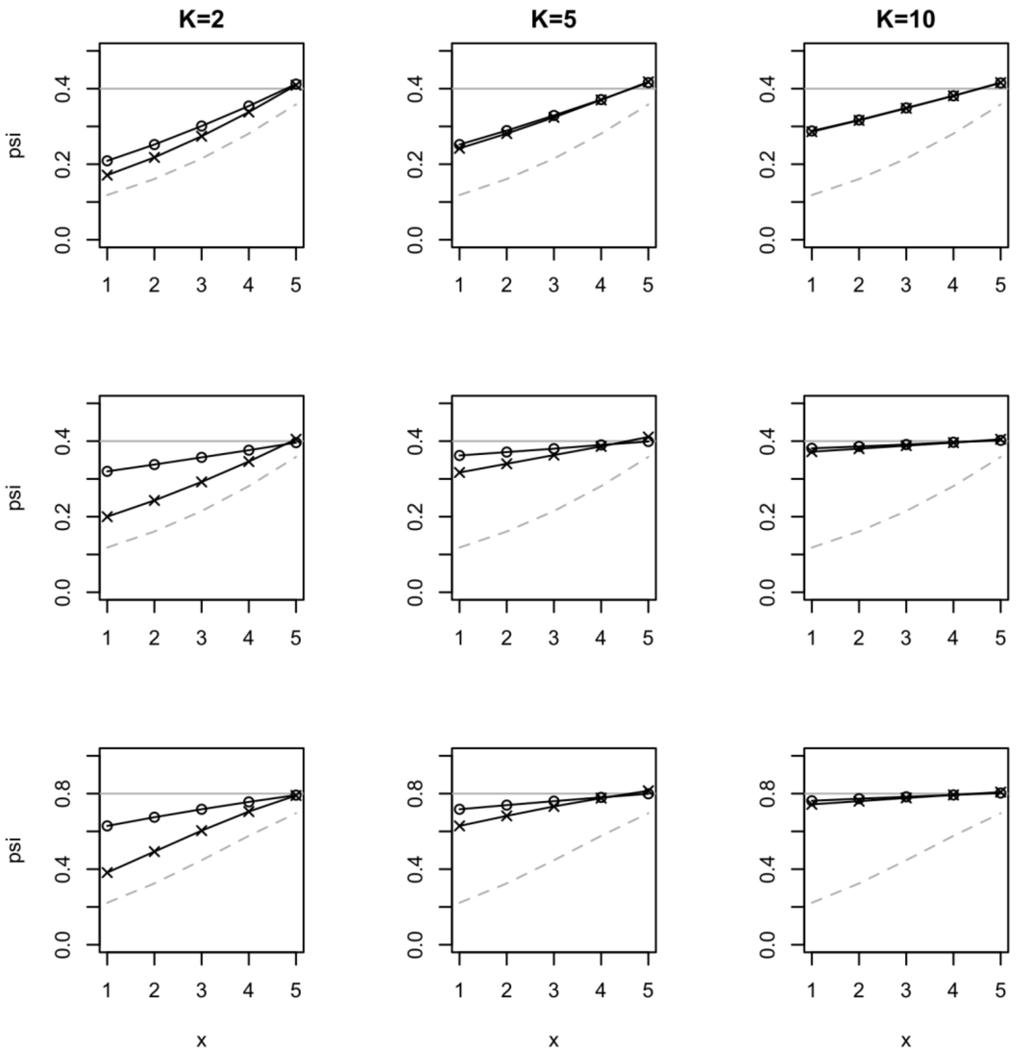

Figure S3.1 Occupancy probabilities as a function of covariate x based on the estimates in Table S3.1 (circles: hierarchical model; crosses: naïve model). The grey line shows the actual occupancy level. Each row corresponds to one scenario (B1 to B3). Dash grey lines represent results obtained with the naïve model when only one survey worth of effort is applied to each site (i.e. $K = 1$).
